# Two New Cynodonts (Therapsida) from the Middle-Early Late Triassic of Brazil and Comments on South American Probainognathians

**DOI:** 10.1371/journal.pone.0162945

**Published:** 2016-10-05

**Authors:** Agustín G. Martinelli, Marina Bento Soares, Cibele Schwanke

**Affiliations:** 1 Laboratório de Paleontologia de Vertebrados, Departamento de Paleontologia e Estratigrafia, Instituto de Geociências, Universidade Federal do Rio Grande do Sul (UFRGS), Ave. Bento Gonçalves 9500, Agronomia, 91540–000, Porto Alegre, RS, Brazil; 2 Instituto Federal de Educação, Ciência e Tecnologia do Rio Grande do Sul, Campus Porto Alegre, Rua Coronel Vicente 281, Centro Histórico, 90030–040, Porto Alegre, RS, Brazil; Ecole normale superieure de Lyon, FRANCE

## Abstract

We describe two new cynodonts from the early Late Triassic of southern Brazil. One taxon, *Bonacynodon schultzi* gen. et sp. nov., comes from the lower Carnian *Dinodontosaurus* AZ, being correlated with the faunal association at the upper half of the lower member of the Chañares Formation (Ischigualasto-Villa Unión Basin, Argentina). Phylogenetically, *Bonacynodon* is a closer relative to *Probainognathus jenseni* than to any other probainognathian, bearing conspicuous canines with a denticulate distal margin. The other new taxon is *Santacruzgnathus abdalai* gen. et sp. nov. from the Carnian *Santacruzodon* AZ. Although based exclusively on a partial lower jaw, it represents a probainognathian close to *Prozostrodon* from the *Hyperodapedon* AZ and to *Brasilodon*, *Brasilitherium* and *Botucaraitherium* from the *Riograndia* AZ. The two new cynodonts and the phylogenetic hypothesis presented herein indicate the degree to which our knowledge on probainognathian cynodonts is incomplete and also the relevance of the South American fossil record for understanding their evolutionary significance. The taxonomic diversity and abundance of probainognathians from Brazil and Argentina will form the basis of deep and complex studies to address the evolutionary transformations of cynodonts leading to mammals.

## Introduction

Middle to Upper Triassic fossil vertebrate assemblages of South America are well documented in Brazil and Argentina [1–7]. They constitute one of the best vertebrate faunal successions in the world with a wide diversity of temnospondyls, procolophonians, basal lepidosauromorphs and rhynchocephalians, stem turtles, several archosauromorph groups (e.g., rhynchosaurs, basal archosauriforms, pseudosuchians and ornithodirans), and dicynodont and cynodont therapsids preserved [4, 6–12]. In particular, the cynodonts include a wide spectrum of herbivorous/omnivorous traversodontids and carnivorous/omnivorous non-mammaliaform probainognathians [5, 6, 13]. The record of South American probainognathians includes about 20 species, several of them based upon relatively well-known specimens, plus some records tentatively referred to previously known taxa that likely would enlarge this number after new discoveries and studies [8, 11, 14–31].

The oldest putative South America record of a probainognathian comes from the Anisian-Ladinian Cerro de Las Cabras Formation ([32–33] although, it was originally referred to the Río Mendoza Formation, see [34–35]), Cuyana Basin, at Mendoza province, western Argentina. It consists of both lower jaws with dentition ascribed to *Cromptodon mamiferoides*. This species was originally classified as a non-eucynodontian galesaurid [32]. Later on, *Cromptodon* was compared with *Aleodon brachyrhamphus*, from the Middle Triassic of Tanzania and Namibia [36–37], and both species were included within Chiniquodontidae [38–39]. At present, the taxonomic status of *Cromptodon* remains uncertain [15].

The most conspicuous probainognathian record from Argentina comes from the Ischigualasto-Villa Unión Basin. It includes *Chiniquodon theotonicus* [15, 40] and *Probainognathus jenseni* [41] from the lower Upper Triassic Chañares Formation; *Chiniquodon sanjuanensis* ([15], = *Probelesodon sanjuanensis* sensu [6, 23]), *Ecteninion lunensis* [21], *Diegocanis elegans* [22], cf. *Probainognathus* sp. [42], but see [43], and cf. *Chiniquodon sp*. (it was synonymized with *C*. *sanjuanensis* by Abdala and Giannini [15], but Martínez et al., [6] maintained the original proposal of Bonaparte [44]) from the Upper Triassic Ischigualasto Formation; and *Chaliminia musteloides* [25, 45] and cf. *Tritylodon* [8] from the Upper Triassic Los Colorados Formation [46]. In addition, Martínez et al., [47] recently recognized tritheledontid cynodonts within a new faunal association at the Upper Triassic Quebrada del Barro Formation, Marayes-El Carrizal Basin.

**Fig 1:**
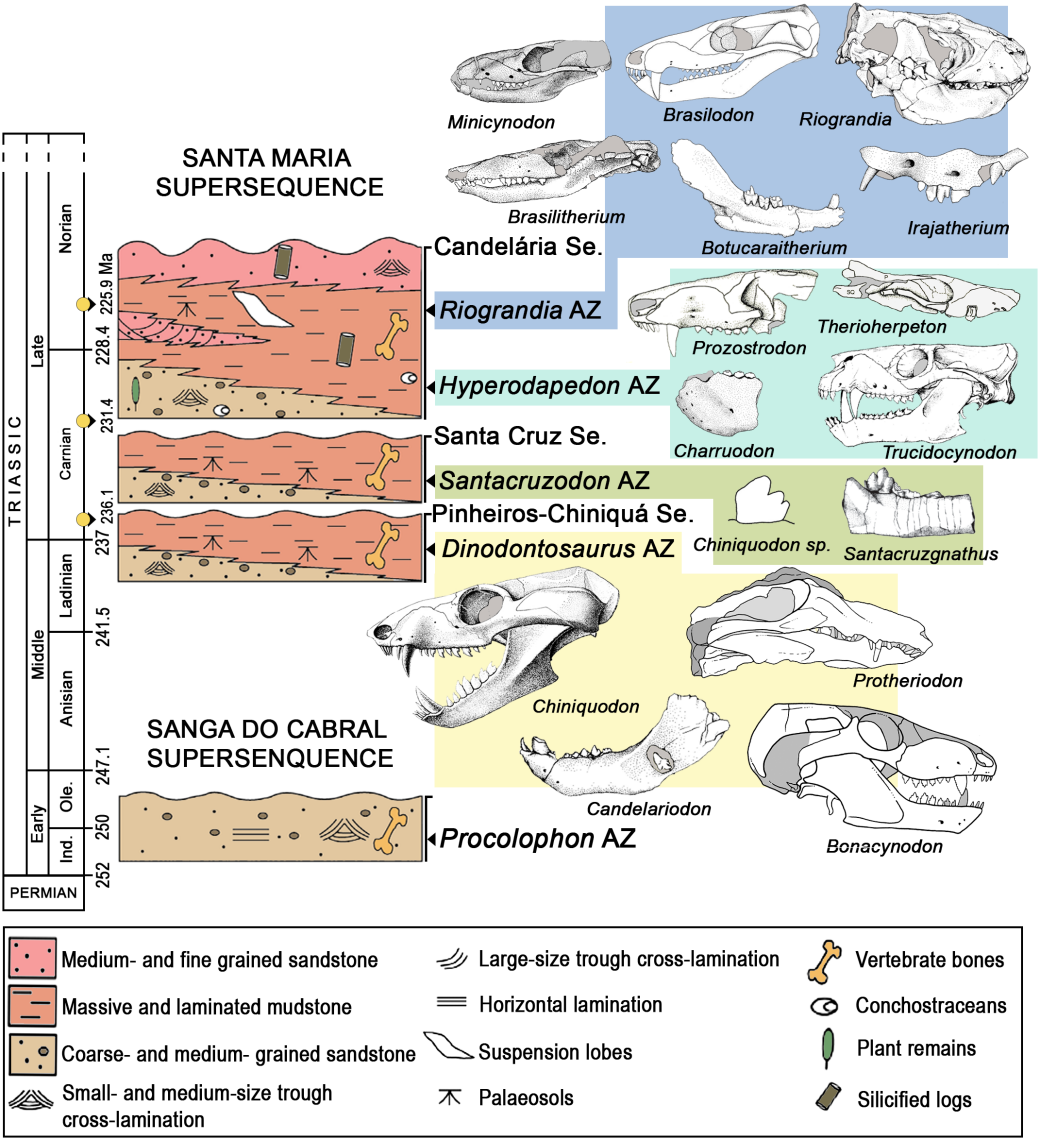
Chrono- and biostratigraphy of Triassic units with vertebrate assemblages zones (AZ) from southern Brazil. Modified from [29, 48, 49]. The ages of the column follow Gradstein et al., [50]. The radiometric dating of 236.1, 231.4 and 225.9 Ma correspond to the first half of the Chañares Formation [51], the base of the Ischigualasto Formation, and the base of Los Colorados Formation [6], respectively. Abbreviations: Ind., Induan; Ole., Olenekian; Se., sequence.

In Brazil, probainognathians are first documented in the Pinheiros-Chiniquá Sequence of the Santa Maria Supersequence [46, 48–51] (Fig 1). This newly recognized sequence corresponds in part to the Santa Maria 1 Sequence of Zerfass et al. [48] and belongs to the lower section of the traditional Santa Maria Formation [52]. At present only one vertebrate assemblage zone (AZ) is recognized within this sequence: the *Dinodontosaurus* AZ. Its probainognathian content includes *Chiniquodon theotonicus* [15, 53], *Candelariodon barberenai* [27], and *Protheriodon estudianti* [20] (Fig 1). Subsequently, the Santa Cruz Sequence [49] includes the *Santacruzodon* AZ, with few probainognathian records, including a solely specimen referred to cf. *Probainognathus* ([30], but see below) and a few specimens referred to *Chiniquodon* sp. [54] (Fig 1). In the overlying Candelária Sequence [49], which corresponds to the Santa Maria 2 Sequence of Zerfass et al. [48] and belongs to the upper part of the traditional Santa Maria Formation [52] and part of the Caturrita Formation [55], two AZs are recognized (Fig 1): the *Hyperodapedon* (older) and *Riograndia* (younger) AZs. The former AZ includes the probainognathians *Trucidocynodon riograndensis* [26], *Therioherpeton cargnini* [16, 56–57], *Charruodon tetracuspidatus* [14], and *Prozostrodon brasiliensis* [16]. The *Riograndia* AZ includes the probainognathians *Brasilodon quadrangularis*, *Brasilitherium riograndensis* [18, 19, 58], *Minicynodon maieri* [11, 58], *Botucaraitherium belarminoi* [31], *Riograndia guaibensis* [17, 29], and *Irajatherium hernandezi* [24, 28] (Fig 1).

As previously noted, the diversity of Middle to Late Triassic South American probainognathian cynodonts is extraordinary and they form the basis of understanding the evolutionary transformations that occurred prior to the establishment of the Mammaliaformes clade [18, 19, 25, 26, 46, 58–70]. In recent years there was a considerable growth in the number of studies that address these issues [67, 68, 70–75]. Nonetheless, several taxonomic, anatomical and phylogenetic aspects of South American probainognathian cynodonts remain poorly explored and further studies are needed. In addition, the diversity of probainognathian cynodonts still remains low, especially in pre-Norian times, when compared with other components of the vertebrate fauna (e.g., [4, 6]).

In this contribution, two new probainognathian cynodonts from southern Brazil are described. One cynodont is based on two specimens discovered by Dr. Llewellyn Ivor Price in the 1940s at the municipality of Candelária, state of Rio Grande do Sul. The two specimens come from the *Dinodontosaurus* AZ of the Pinheiros-Chiniquá Sequence (Fig 1). The other new cynodont taxon is based on the restudy of the specimen referred as cf. *Probainognathus* [30] from the *Santacruzodon* AZ (Fig 1). Both new taxa are analyzed phylogenetically and show the high tooth morphology diversity among Middle-early Late Triassic carnivores/omnivores cynodonts from western Gondwana. Finally, we comment on the anatomy and/or systematic position of other Middle-Late Triassic South American probainognathians (*Probainognathus* and *Protheriodon*).

## Materials and Methods

### Access to specimens

The specimens described here and those used for comparison belong to public collections and were examined with the explicit permission of appropriate curators and/or collection managers (see Acknowledgments). Repository locations and abbreviations for all specimens cited in the text are listed below. We followed all Brazilian regulations for fossil studies and we complied with the PLoS Paleontological Ethics Statement. In Table 1 we listed selected cynodont species and specimens here described, used for comparisons and/or for evaluating character-state assignments in the data matrix.

**Table 1:** List of cynodonts and specimens. Specimens for each taxon that was described, used for comparisons and/or for check character-states in data matrix.

Taxon	Specimens
*Procynosuchus delaharpeae*	AMNH 8220
*Ecteninion lunensis*	PVSJ 422
*Trucidocynodon riograndensis*	UFRGS-PV-1051-T
*Chiniquodon theotonicus*	PVL 4167, 4444, 4674; MCP-PV 1600
*Chiniquodon sanjuanensis*	PVSJ 411; PVL 2076, 2077
*Probainognathus jenseni*	PVL 4169, 4673, 4677, 4678, 4445, 4446, 4447; MCZ 4279, 4295
Cf. *Probainognathus*	PVSJ 410
*Candelariodon barberenai*	MMACR-PV-0001-T
*Protheriodon estudianti*	UFRGS-PV-0962-T
*Kayentatherium wellesi*	MCZ 8812
*Riograndia guaibensis*	UFRGS-PV-0596T, 0601-T, 0624-T, 0623-T, 0788-T, 0833T
*Irajatherium hernandezi*	UFRGS-PV-0599-T, 1068-T
*Chaliminia musteloides*	PVL 3857, PULR 081
*Pachygenelus monus*	SAM 1315, 1329, 1394
*Prozostrodon brasiliensis*	UFRGS-PV-0248-T
*Botucaraitherium belarminoi*	MMACR-PV-003-T
*Brasilitherium riograndensis*	UFRGS-PV-0795-T, 0693-T, 0795-T, 0785-T, 0786-T, 0804-T, 0834-T, 0929-T, 1043-T
*Brasilodon quadrangularis*	UFRGS-PV-0611-T, 0595-T, 0765-T
*Minicynodon maieri*	UFRGS-PV-1030-T
*Bonacynodon schultzi*	MCT-1716-R, 1717-R
*Santacruzgnathus abdalai*	UFRGS-PV-1121-T
*Morganucodon oehleri*	CUP 2320

### Materials

**Fig 2:**
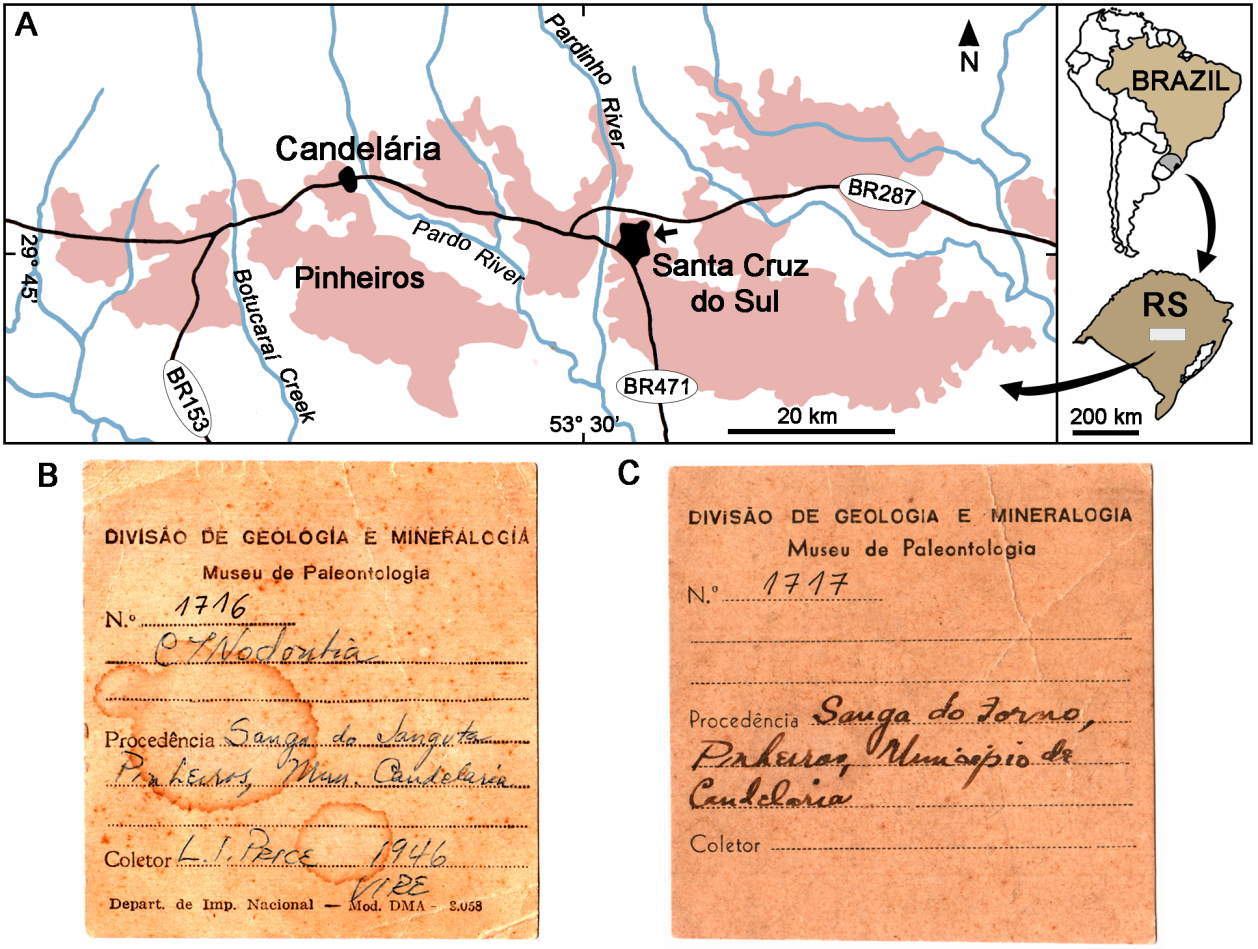
Localities and Catalog cards. (A) Map showing the Middle to Upper Triassic outcrops (in light red colour) in Candelária and Santa Cruz do Sul municipalities, state of Rio Grande do Sul (RS), southern Brazil (based partially on [80]). The two specimens (MCT-1716-R and MCT-1717-R) of *Bonacynodon* come from the Pinheiros region, about 12 km south of Candelária city, and the arrow indicates the site where the specimen (UFRGS-PV-1121-T) of *Santacruzgnathus* was discovered from the *Santacruzodon* AZ. Catalog cards of MCT-1716-R (holotype) from “*Sanga do Janguta*” (B) and MCT-1717-R (referred specimen) from “*Sanga do Forno*” (C) of *Bonacynodon schultzi*.

The first part of this contribution consists on the analysis of two new specimens (MCT-1716-R and MCT-1717-R) that represent a new genus and species. They were found in the Pinheiros district, municipality of Candelária, state of Rio Grande do Sul, southern Brazil (Fig 2). This region has several outcrops belonging to the *Dinodontosaurus* AZ, with a particularly rich fossil record that includes procolophonians, dicynodonts, traversodontids, probainognathians, proterochampsids, and rauisuchians [4, 10, 76–78].

The new specimens have no a specific location other than the name of the *sanga* in which they were discovered. The term *sanga* (*sangas* in plural) was commonly used since the works of Friedrich von Huene ([53]; there is a summary about the usage of this word in [79]). A *sanga* likely represents a relatively small bad-land outcrop, which was originated mainly by water erosion and is partially covered with vegetation (mainly at the top and at the bottom). The *sanga* is usually located close to an artificial lagoon where the water is gathered. Over the time, the term *sanga* was also used to mention small bad-land outcrops that are not necessary associated to water courses. The *sangas* change their physiognomy significantly over time due to continuous erosion and are also intensely affected by human activities; therefore, several historical fossiliferous *sangas* disappeared [79] or simply the exact location is unknown [4].

The catalog cards refer the specimen MCT-1716-R to the “*Sanga do Janguta*” and MCT-1717-R to “*Sanga do Forno*” (Fig 2). According to historical data, there was a limestone burning furnace in the area known as “*Sanga dos Fósseis*” (Fossil´s *Sanga*) or “*Sanga Pinheiros*”. Therefore, the “*Sanga do Forno*” (Furnace´s *Sanga*) would likely correspond to the “*Sanga dos Fósseis*/*Sanga Pinheiros*”. Both specimens were collected by L. I. Price at the time he worked for the *Departamento Nacional de Produção Mineral* (DNPM) of Rio de Janeiro, with MCT-1716-R being found in 1946 (Fig 2). Both specimens can be referred unambiguously to the same taxon. They share the same skull and tooth morphology, a similar size, and come from the same AZ and region.

The holotype MCT-1716-R was found together with an articulated skeleton of a mid-sized *Dinodontosaurus* (specimen MCT-319-R) [81]. The skull was removed during preparation and the partial skeletal remains rest on the *Dinodontosaurus* left femur [81]. Unfortunately, the postcranium is extremely poorly preserved, hampering the observation of any anatomical information.

Schwanke and Kellner [81] suggested that the association of this cynodont together with the articulated skeleton of *Dinodontosaurus* is likely indicative of an opportunist necrophagian behavior of the former. To this example, they also added the case of the probainognathian *Therioherpeton cargnini*, which was also found in association with an articulated skeleton of the rhynchosaur *Hyperodapedon* [56]. The taphonomic evidence at present is limited and does not conclusively support such a hypothesis. For example, the small-sized probainognathians could have been eating invertebrates (larvae, worms, etc.) present at the decaying dead animal.

The other part of this contribution includes the re-analysis of a partial lower jaw (UFRGS-PV-1121-T) from the *Santacruzodon* AZ (Fig 1) that was referred to cf. *Probainognathus* [30]. We propose a new genus and species and provide a diagnosis for this taxon, *Santacruzgnathus abdalai* gen. et sp. nov., due to the presence of a unique combination of features.

Absolute dates at the Chañares and Ischigualasto formations (Ischigualasto-Villa Unión Basin, Argentina) [6, 7, 9, 51], a preliminary dating at the *Santacruzodon* AZ [82], and tetrapod faunal correlations suggest that the *Dinodontosaurus* AZ from southern Brazil (correlated with the faunal association at the upper half of the lower member of the Chañares Formation; see [83]) is early Carnian in age (Fig 1) and the *Santacruzodon* AZ is entirely included within the Carnian.

### Phylogenetics

A phylogenetic analysis was conducted based on 36 species and 145 morphological cranial, dental and postcranial characters (S1 File). The data matrix used here is based on that presented by Liu and Olsen [71], which in turn was constructed using previous analyses on cynodont relationships, such as Hopson and Barghusen [84], Rowe [85], Wible [86], Crompton and Luo [87], Luo and Crompton [62], Luo [61], Martínez et al. [21], Luo et al. [88], Hopson and Kitching [63], Abdala and Ribeiro [89], Bonaparte et al. [18, 19], Martinelli et al. [24], Abdala [90], and Martinelli and Rougier [25]. To this data matrix we introduced modifications on characters and codifications, a few of them were already included in the phylogenetic analysis of Soares et al. [31].

In order to enlarge the sample of South American probainognathian cynodonts, we added the following terminal species: *Protheriodon estudianti* [20], *Brasilitherium riograndensis* [11, 18, 19, 58], *Botucaraitherium belarminoi* [31] and *Bonacynodon schultzi* (this study). Liu and Olsen [71] considered *Brasilodon* and *Brasilitherium* as synonyms; therefore, they used only *Brasilodon* as a terminal unit. The large sample of specimens of *Brasilodon* and *Brasilitherium* (in addition to the close relative *Minicynodon*) [11] is being further analyzed by the authors (AGM and MBS) in order to explore the synonymy and taxonomy of these taxa. As stated by previous authors [72, 71], the synonymy of both taxa is likely appropriate. Because the holotype of *Brasilodon* is a partial skull without lower jaws and some diagnostic features among brasilodontids are in the lower dentition, we have considered *Brasilodon* and *Brasilitherium* as separate terminal units for the current analyses, as proposed originally by Bonaparte et al. [10, 18, 19, 59].

The data matrix (see Supporting Information) was managed with NEXUS Data Editor (NDE) [91]. The matrix was analyzed using Maximum Parsimony with equally weighted characters with the computer program TNT 1.1 (New Technology Search) [92]. All characters were treated as non-additive. The equally weighted parsimony analysis was conducted performing a heuristic search of Wagner trees with 1000 random addition sequences, followed by TBR (Tree Bisection Reconnection), and saving 10 trees per round.

Unless otherwise noted, clade names follow the phylogenetic definitions by Hopson and Kitching [63] and Liu and Olsen [71].

### Nomenclatural acts

The electronic edition of this article conforms to the requirements of the amended International Code of Zoological Nomenclature, and hence the new names contained herein are available under that Code from the electronic edition of this article. This published work and the nomenclatural acts it contains have been registered in ZooBank, the online registration system for the ICZN. The ZooBank LSIDs (Life Science Identifiers) can be resolved and the associated information viewed through any standard web browser by appending the LSID to the prefix http://zoobank.org/. The LSID for this publication is: urn:lsid:zoobank.org:pub:3A96FA8E-37BA-4730-8471-1A7DF9ED739D. The electronic edition of this work was published in a journal with an ISSN, and has been archived and is available from the following digital repositories: PubMed Central and LOCKSS.

### Institutional abbreviations

AMMH, American Museum of Natural History (New York, USA); CUP, Catholic University of Peking (Peking, China; specimen observed at the Department of Organismal Biology, University of Chicago, Chicago, USA); MCP-PV, Museu de Ciências e Tecnologia (Paleontological Collection), Pontifícia Universidade Católica do Rio Grande do Sul (Porto Alegre, Brazil); MCZ, Museum of Comparative Zoology, Harvard University (Cambridge, Massachusetts, USA); MMACR-PV-T, Museu Municipal Aristides Carlos Rodrigues, Paleovertebrates-Triassic Collection (Candelária, Rio Grande do Sul, Brazil); MCT, Museu de Ciências da Terra (Rio de Janeiro, Brazil); PULR, Museo de Antropología of the Universidad Nacional de La Rioja (La Rioja, Argentina); PVL, Instituto Miguel Lillo (Vertebrate Paleontology Collection) of the Universidad Nacional de Tucumán (San Miguel de Tucumán, Argentina); PVSJ, Vertebrate Paleontology, Universidad Nacional de San Juan (San Juan, Argentina); SAM, Iziko South African Museum (Cape Town, South Africa; specimen observed at the Department of Organismal Biology, University of Chicago, Chicago, USA); UFRGS-PV-T, Universidade Federal do Rio Grande do Sul, Vertebrate Paleontology, Triassic Collection (Porto Alegre, Brazil).

### Anatomical abbreviations

A/a, B/b, C/c, D/d, upper/lower cusps on postcanines; ang, angular; art+pra, articular plus prearticular; bo, basioccipital; bs, basisphenoid; ca, cavum epiptericum; C/c, upper/lower canine; ch, choana; co, coronoid bone; cop, coronoid process; d, dentary; dl, groove for dental lamina; dlc, distolingual cingulum; e, epipterygoid; eo, exoccipital; fo, fenestra ovalis; I/i, upper/lower incisor; iof, infraorbital foramen; j, jugal; jf, jugular fossa; l, lacrimal; lc, lambdoidal crest; ld, left dentary; lf, lateral flange; lI, left upper incisor; lpc, last postcanine; mf, masseteric fossa; Mg, Meckelian groove; mlc, mesiolingual cingulum; mx, maxilla; o, orbit; or, orbital rim; op/pr, ophistotic/prootic; PC/pc, upper/lower postcanine; pcr, parietal crest; pdc, postdentary complex; pdt, postdentary trough; pl, palatine; pm, premaxilla; po, postorbital; pp, paroccipital process; pt, pterygoid; qr, quadrate ramus of the epipterygoid; r, root; rC, replacement upper canine; rI, right upper incisor; rlam, reflected lamina of the angular; s, symphysis; sep, secondary palate; sp, splenial; sq, squamosal; sur, surangular; t, tabular.

## Results

Below two new taxa are described: *Bonacynodon schultzi* gen. et sp. nov. and *Santacruzgnathus abdalai* gen. et sp. nov.

### Systematic paleontology

Therapsida Broom, 1905

Cynodontia Owen, 1861

Eucynodontia Kemp, 1982

Probainognathia Hopson, 1990

Probainognathidae Romer, 1973

#### Revised definition

The clade including the most recent common ancestor of *Probainognathus jenseni* and *Bonacynodon schultzi*, and all its descendants.

#### Comments

The family Probainognathidae was erected by Romer [93] to include *Probainognathus jenseni* [41]. Originally *Probainognathus* was added within Chiniquodontidae [41, 94, 95] but with the description of *Probelesodon minor*, Romer [93] recognized several differences to split out *Chiniquodon*, *Beleson* and *Probelesodon* (at that time they considered as separated genera) from *Probainognathus*. After the proposal of this family, it was not considered in several contributions (e.g., [96, 97]) and remained monotypic [84]. *Probainognathus jenseni* is presently restricted to the Chañares Formation in western Argentina. Bonaparte and Crompton [42] described a juvenile skull and lower jaws (PVSJ 410) from the Ischigualasto Formation as belonging to cf. *Probainognathus*. A preliminary result of the reanalysis of PVSJ 410 suggests it represents a distinct cynodont taxon (see [42]). From Brazil, Soares et al., [30] described a lower jaw with a posterior postcanine (UFRGS-PV-1121-T) from the *Santacruzodon* AZ as cf. *Probainognathus*. The restudy of this specimen (see below) indicates the jaw belongs to a new probainognathian more derived than *Probainognathus*. Our phylogenetic analysis groups *Probainognathus* and the new taxon here studied (*Bonacynodon schultzi*) as members of Probainognathidae.

#### Diagnosis

Small cynodonts with an skull length of 6–7cm with the following combination of features (autapomorphies with an asterisk*): skull with broad temporal region and temporal region longer than the muzzle*; long secondary palate with the palatine contributing less than the maxilla; large and transversely narrow upper and lower canines; sectorial upper and lower postcanines with tall crown and unconstricted root; postcanine crowns with main cusp a/A, and well-developed cusps b/B, c/C, d/D displaced in line with well-defined groove separating each cusp; small mesiolabial and distolabial cingula (not continuous) in lower postcanines; not curved cusps in postcanines; posterior part of the maxillary tooth row directed toward the center of the suborbital fenestra and ending anterior to the orbit*; horizontal ramus of dentary proportionally tall in comparison to the high of postcanine crowns in juveniles and adults; incipient glenoid fossa in the squamosal for surangular bone ([15, 41, 98, 99], this study).

*Bonacynodon* gen. nov.

urn:lsid:zoobank.org:act:A63C0DF3-578A-4CC8-9C44-3D27F59A79EE

#### Type and only known species

*Bonacynodon schultzi* sp. nov.

#### Diagnosis

*Bonacynodon* is diagnosed with the following association of characters (autapomorphy with an asterisk*): four upper incisors, circular in cross-section; I1 to I3 of similar size, I4 slightly smaller; incisors widely spaced, with largest diastema between I3 and I4, and I4 and canine; upper canine large, narrow labiolingually and elongate mesiodistally, with conspicuous denticles at least on the distal margin*; upper postcanines narrow labiolingually, bearing three to five aligned cusps; anterior three postcanines with three cusps, symmetrical, with main cusp A and accessory cusps B and C; postcanines fourth and fifth the largest of the series, asymmetrical with large main cusp A, small cusp B, and two or three distal accessory cusps (C, D, and accessory); height relationship among cusps A>C≥B>D; last upper postcanines (PC6) reduced, similar in morphology to anterior postcanines; lower postcanines narrow labiolingually, with up to four aligned cusps; prominent main cusp a, with tall cusp b, small cusp c and d in posterior postcanines; height relationship among cusps a>b>c>d; well-developed articular process of the dentary; well-developed masseteric fossa extending forward below last preserved postcanines; unfused mandibular symphysis.

#### Etymology

*Bona*, in honor to the Argentinean paleontologist Dr. José Fernando Bonaparte for his huge contributions to the knowledge of cynodonts and other vertebrates from the Triassic of Brazil and Argentina. *Cynodon*, Cyno- coming from the Ancient Greek κύων (kuōn, "dog"), in reference to its inclusion into Cynodontia, the members with “dog-like teeth”.

*Bonacynodon schultzi* sp. nov.

urn:lsid:zoobank.org:act:BC29AD4C-8143-4623-9769-5A7AB4712E70

#### Holotype

**Fig 3:**
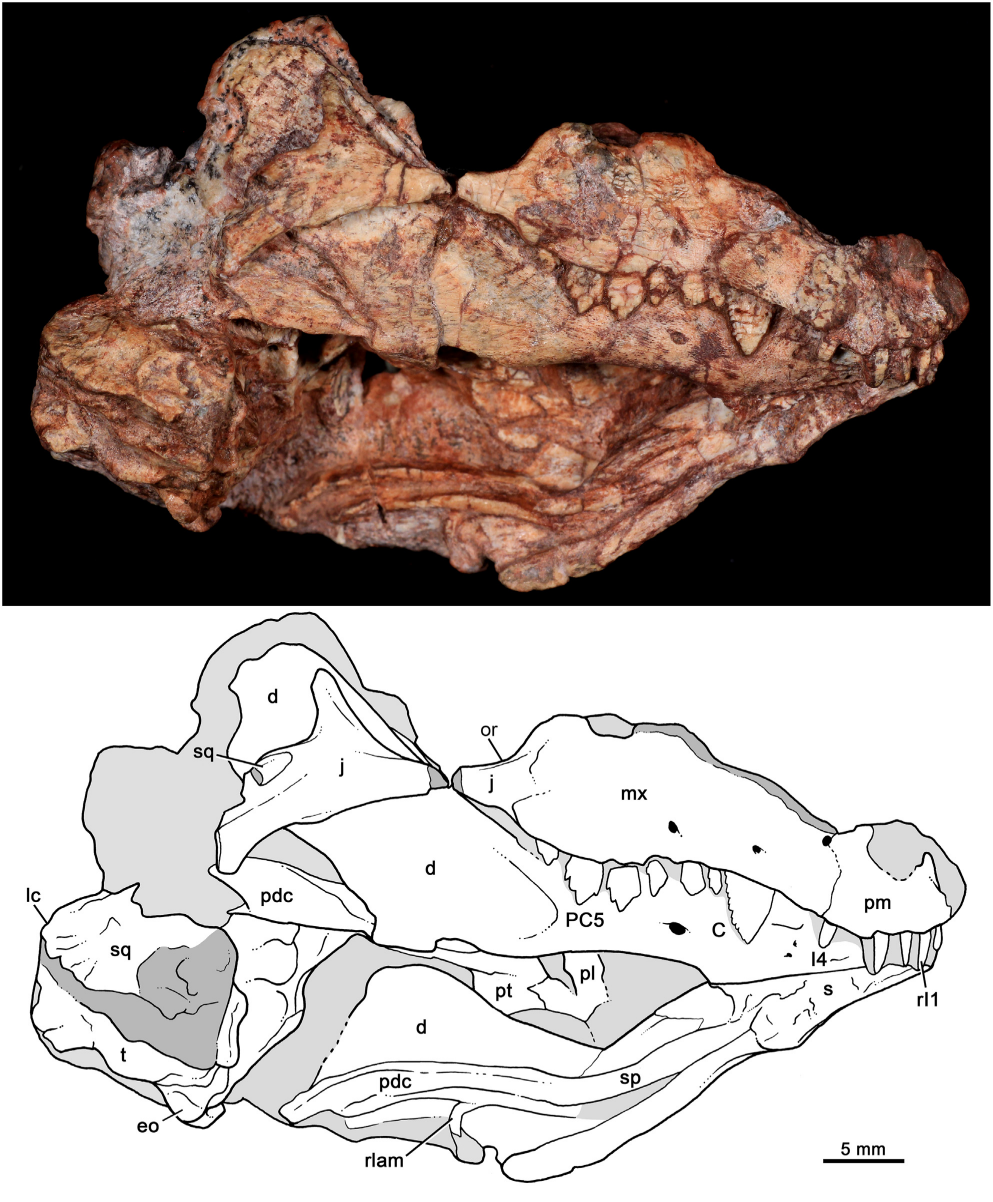
*Bonacynodon schultzi*, holotype MCT-1716-R. Partial skull and lower jaws, with the snout and right lower jaw positioned in lateral view. Dark grey indicates broken areas and soft grey indicates matrix.

**Fig 4:**
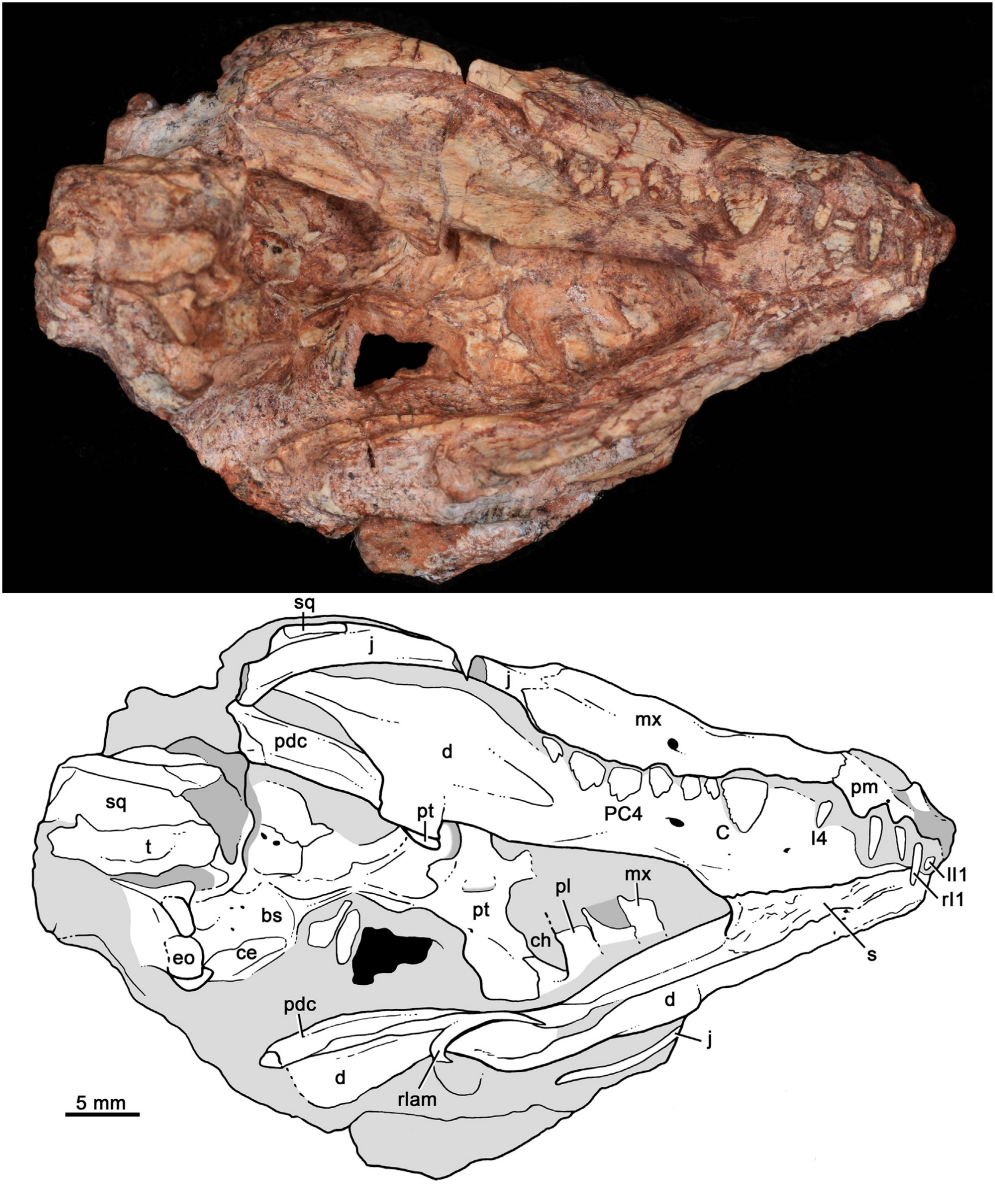
*Bonacynodon schultzi*, holotype MCT-1716-R. Partial skull and lower jaws, with the secondary palate horizontally positioned, in ventral view. Dark grey indicates broken areas and soft grey indicates matrix.

**Fig 5:**
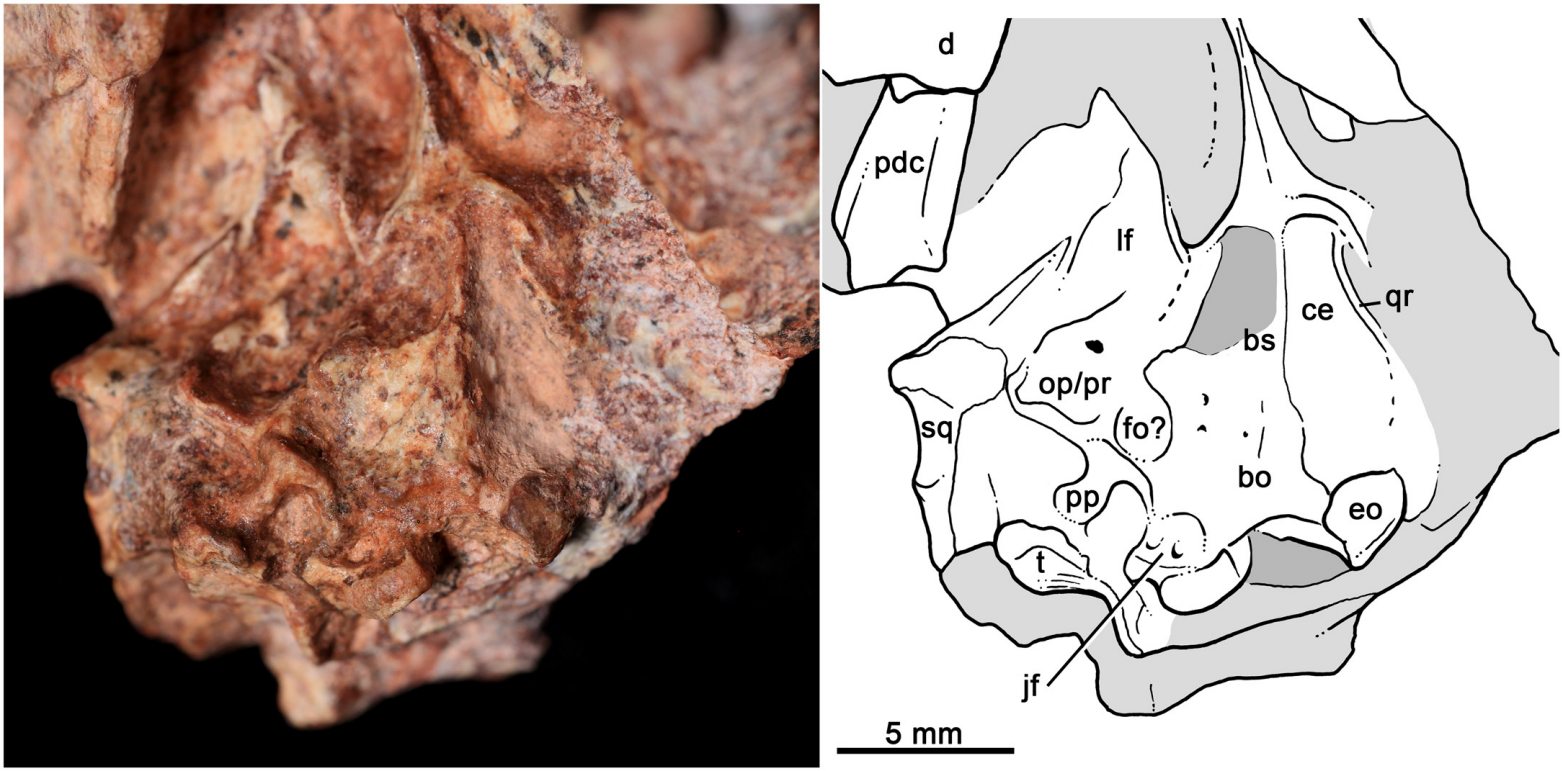
*Bonacynodon schultzi*, holotype MCT-1716-R. Detail of the basicranial region in ventral view with interpretative line drawing.

**Fig 6:**
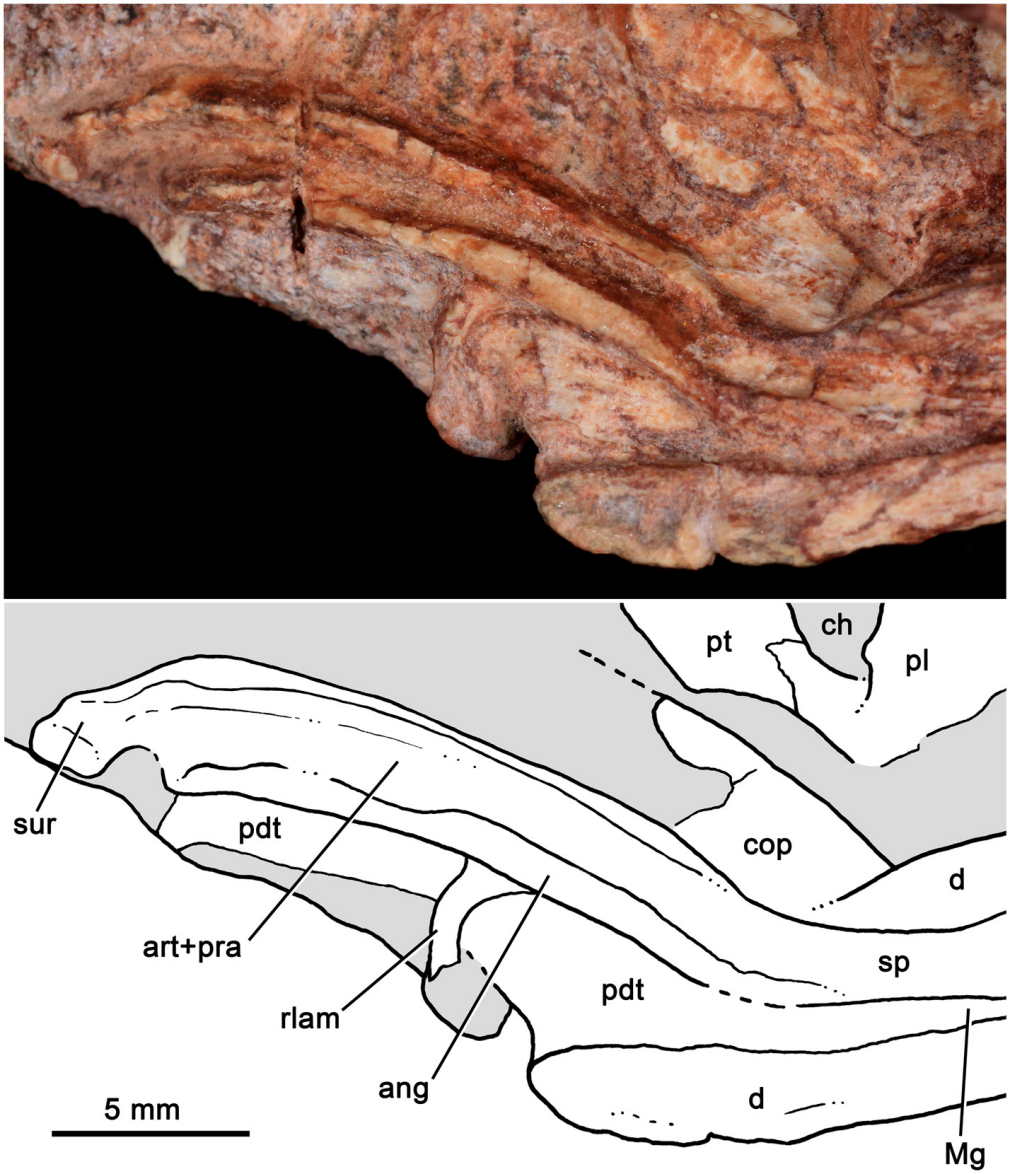
*Bonacynodon schultzi*, holotype MCT-1716-R. Detail of the left postdentary bones in medial view with interpretative line drawing.

**Fig 7:**
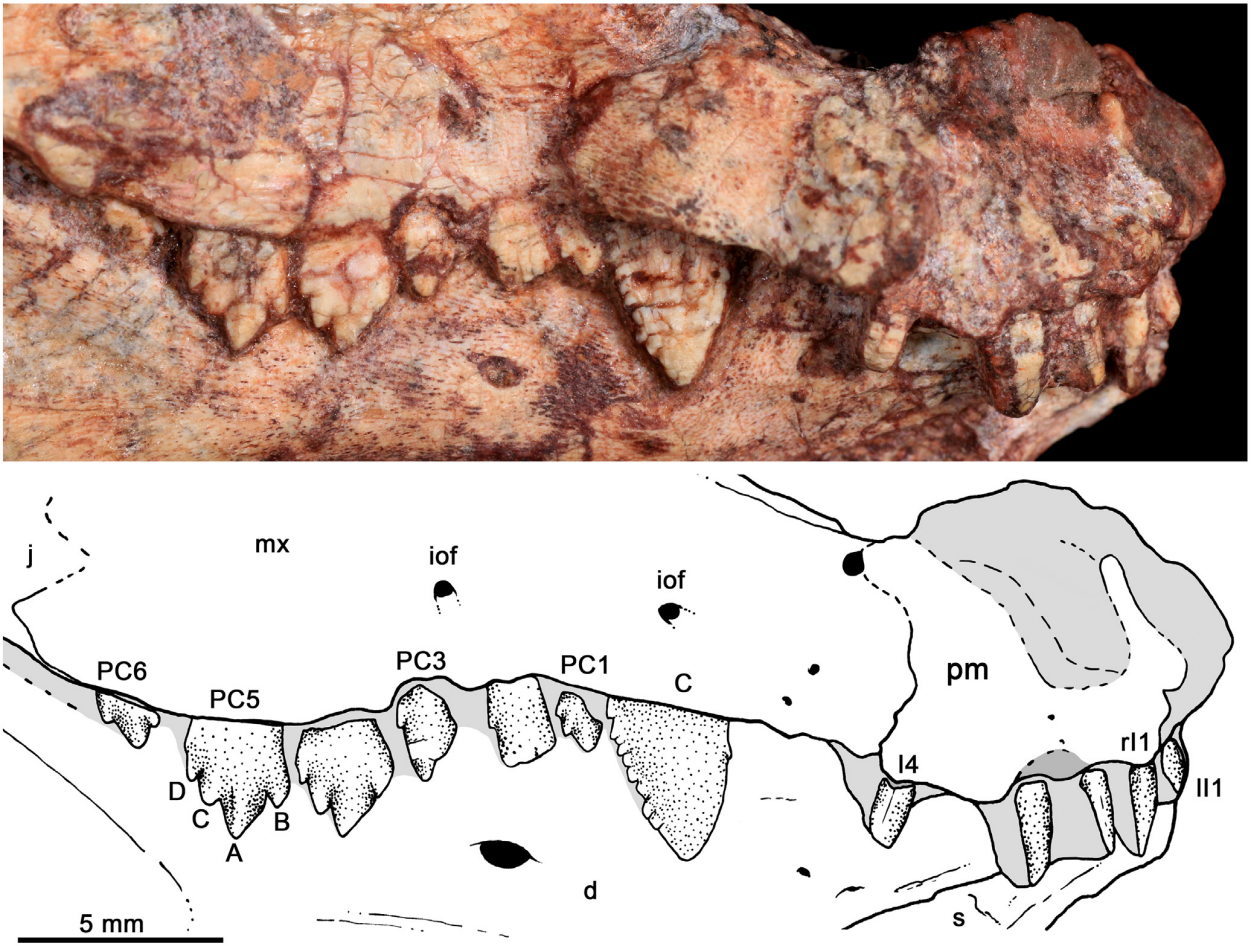
*Bonacynodon schultzi*, holotype MCT-1716-R. Detail of the right upper dentition in lateral view. Dark grey indicates broken areas and soft grey indicates matrix.

**Fig 8:**
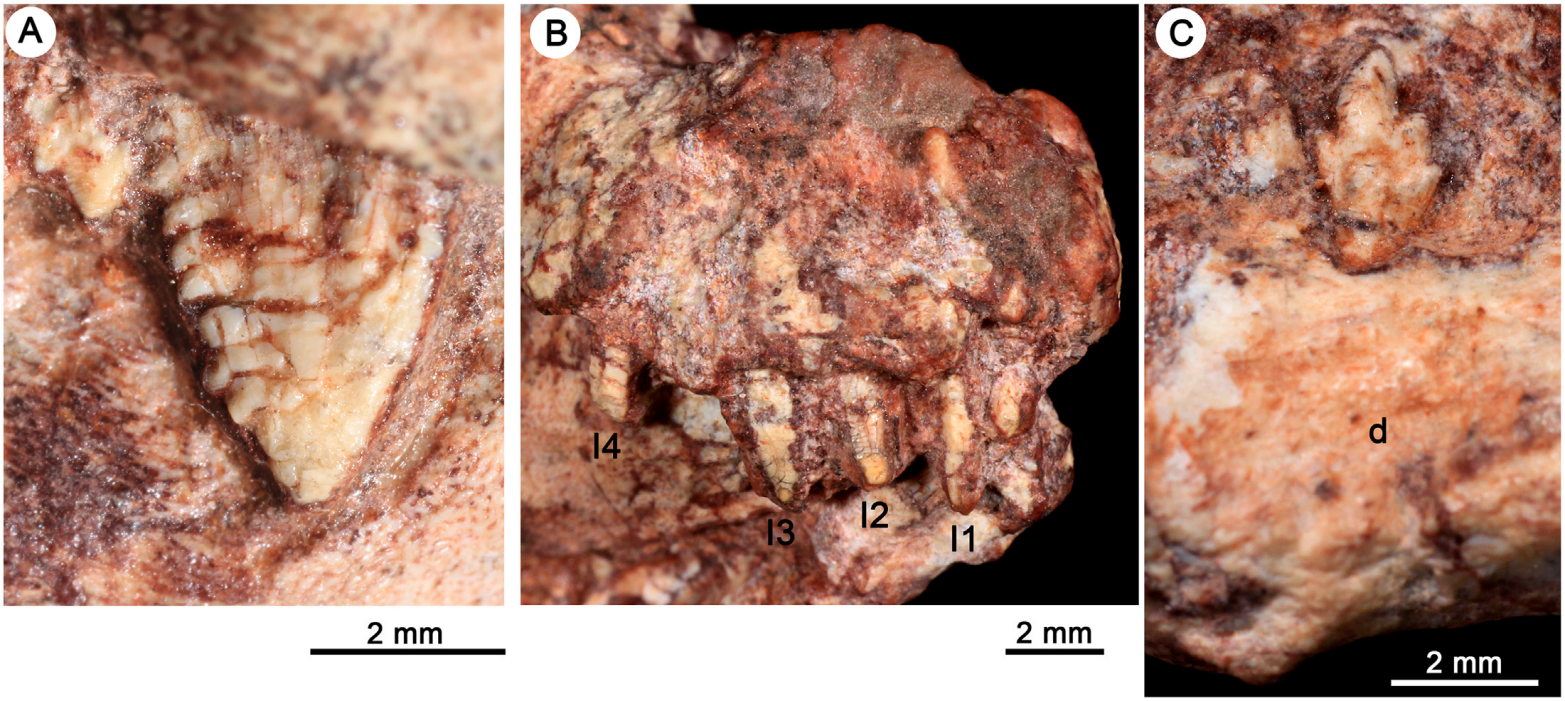
*Bonacynodon schultzi*, holotype MCT-1716-R. Detail of right upper canine in lateral labial view (A). Detail of incisors in anterolateral view (B). Detail of the only lower postcanines of the holotype, two anterior-middle left teeth in labial view (C).

MCT-1716-R, partial skull lacking most of the skull roof with partial dentition, articulated lower jaws, and part of its postcranial skeleton, although poorly preserved (Figs 3–8).

#### Referred specimen

**Fig 9:**
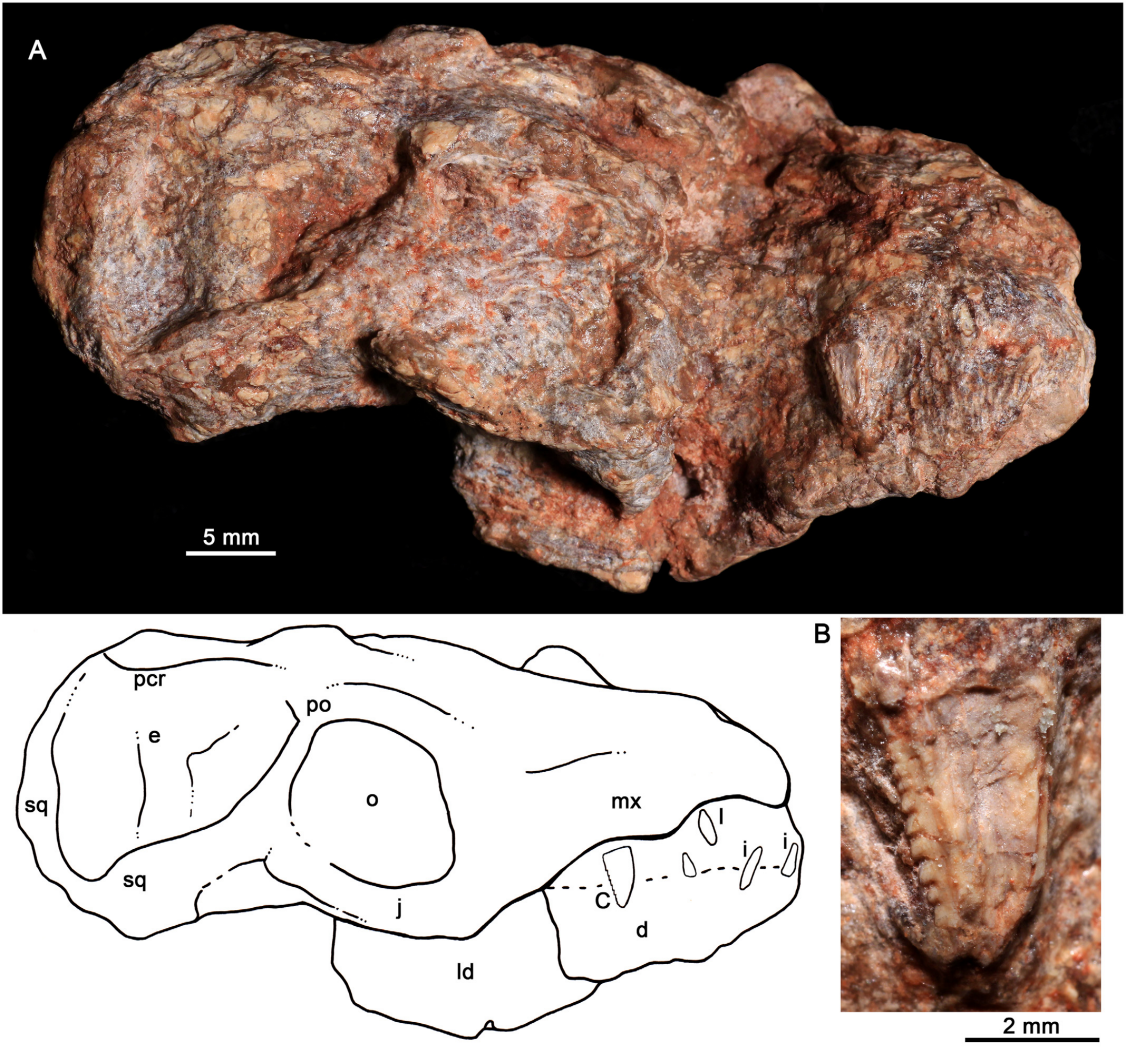
*Bonacynodon schultzi*, referred specimen MCT-1717-R. Partial skull and lower jaws in right laterodorsal view (A) and detail of the right upper canine (B).

**Fig 10:**
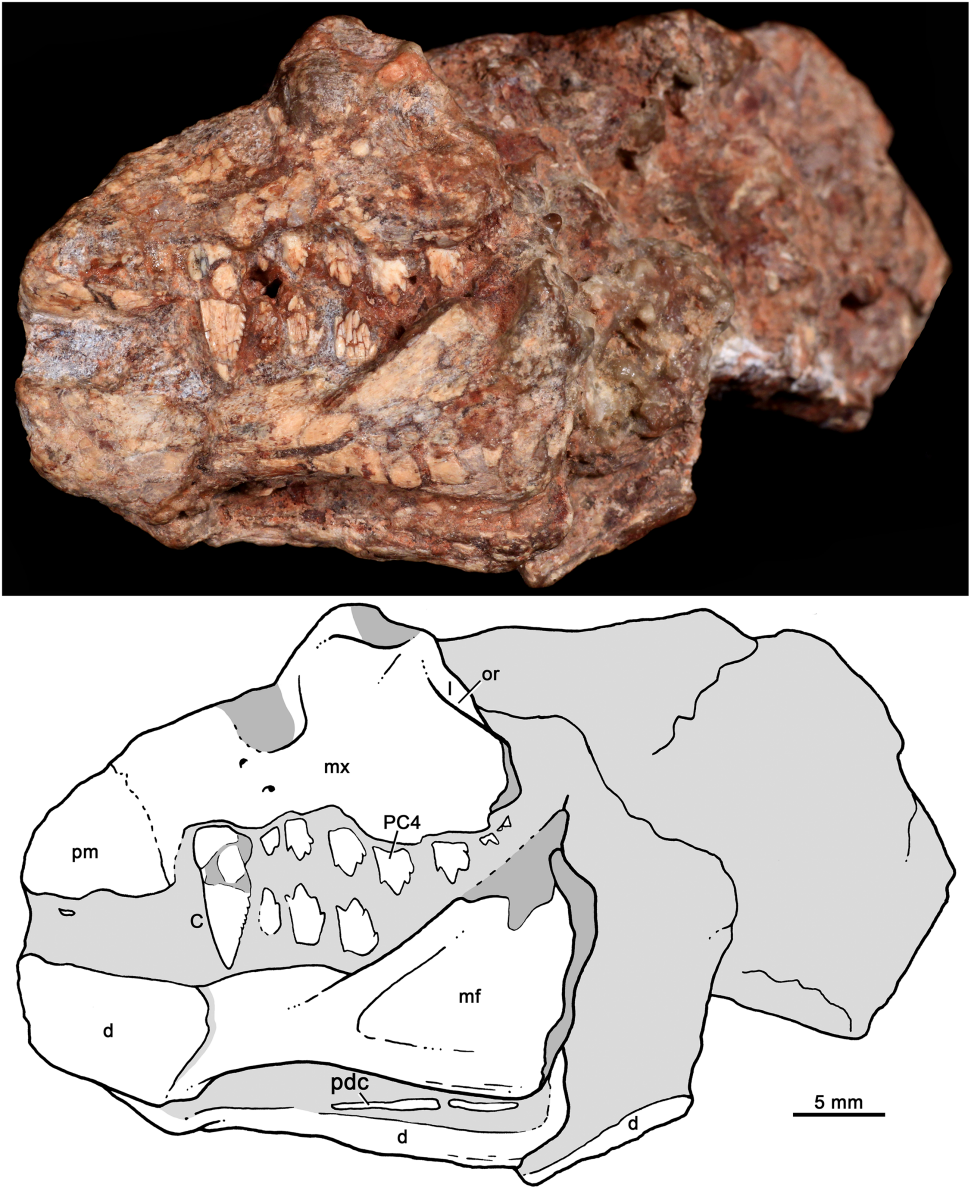
*Bonacynodon schultzi*, referred specimen MCT-1717-R. Partial skull and lower jaws in left lateral view. Dark grey indicates broken areas and soft gray indicates matrix.

**Fig 11:**
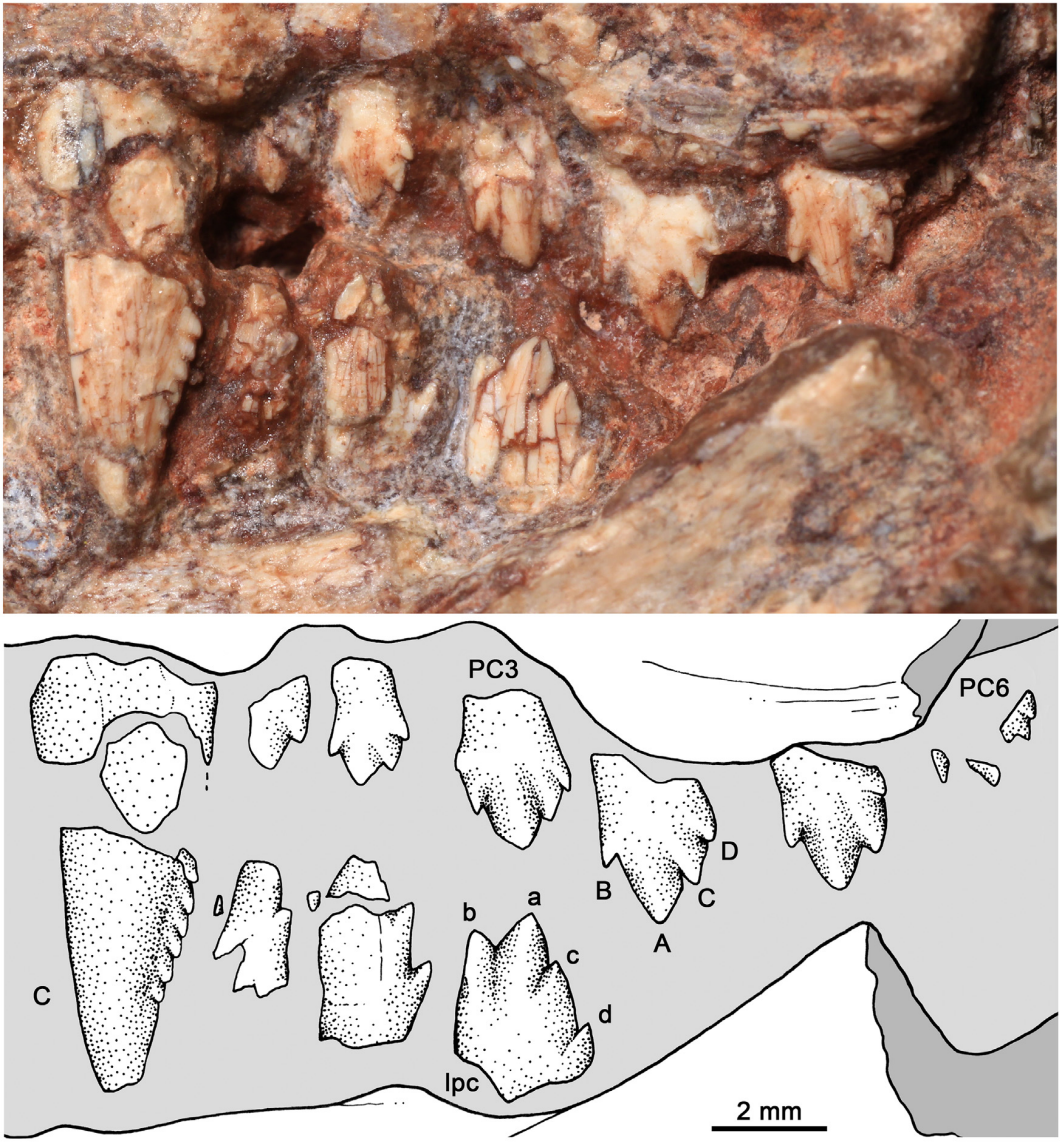
*Bonacynodon schultzi*, referred specimen MCT-1717-R. Detail of upper canine and postcanine dentition in labial view. Dark grey indicates broken areas and soft grey indicates matrix.

**Fig 12:**
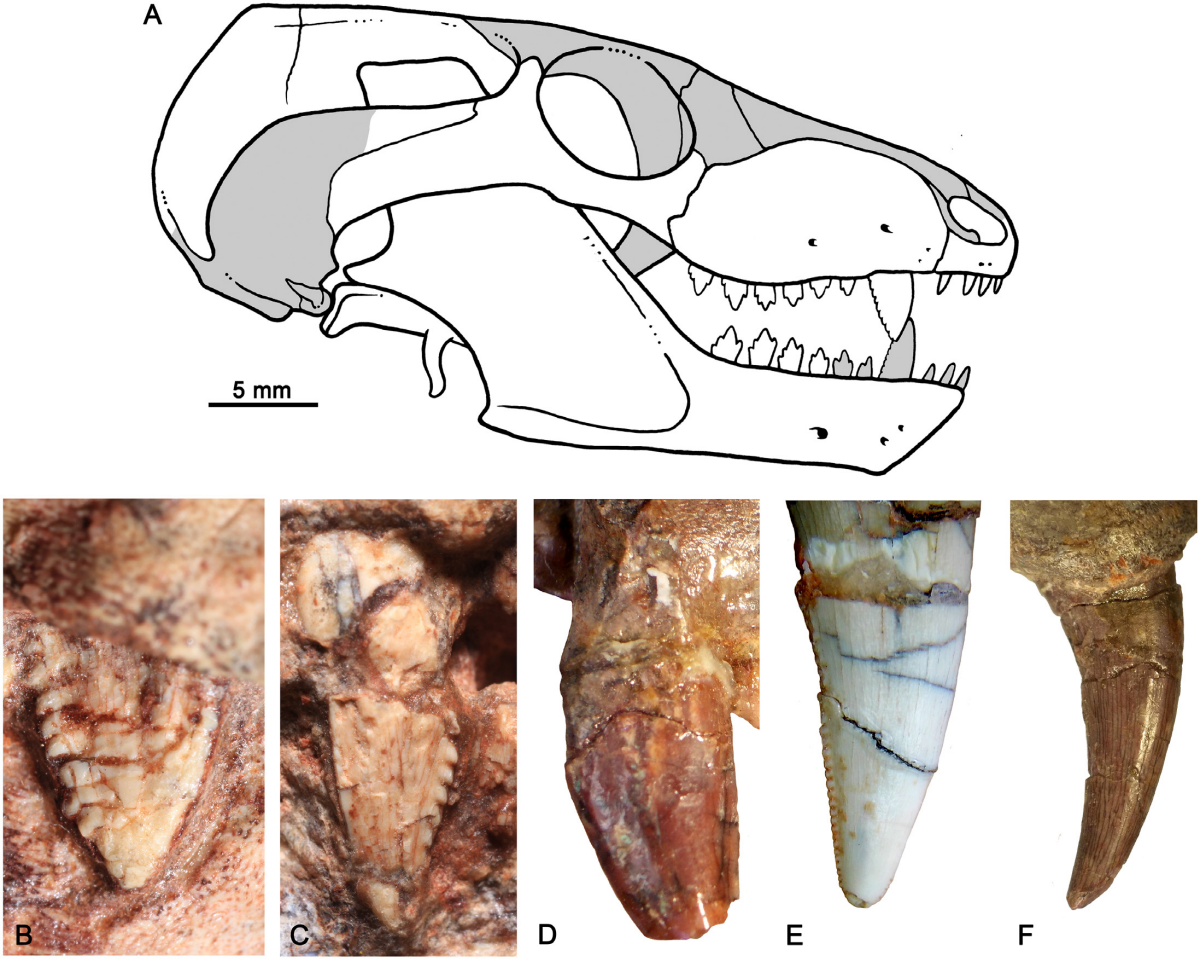
Skull reconstruction and upper canines morphologies. (A) Reconstruction of the skull of *Bonacynodon schultzi*, in lateral view, based on both known specimens and its close relative *Probainognathus jenseni* [41, 63]. Comparisons of upper canines in labial view. (B) right canine of *Bonacynodon* (MCT-1716-R), (C) left canine of *Bonacynodon* (MCT-1717-R), (D) left canine of *Probainognathus* (PVL 4673), (E) apical half of right canine of *Trucidocynodon* (UFRGS-PV-1051-T), and (F) right canine of *Chiniquodon* (PVL 4167). Not to scale.

MCT-1717-R, partial skull with articulated lower jaws, with partial upper and lower dentition (Figs 9–12).

#### Diagnosis

As for genus.

#### Etymology

Species named in honor to Dr. Cesar Leandro Schultz, professor at the Universidade Federal do Rio Grande do Sul (UFRGS, Porto Alegre, Brazil), for his contributions to the paleontology and biostratigraphy of Triassic vertebrate faunas of southern Brazil and for his continuous encouragement and guidance to students at the UFRGS.

#### Locality and horizon

Both specimens come from the Pinheiros region, Candelária municipality, state of Rio Grande do Sul, Brazil. Pinheiros-Chiniquá Sequence [49], Santa Maria Supersequence [48], *Dinodontosaurus* AZ, which is considered early Carnian in age.

### Description and comparisons

Both specimens have suffered post-mortem deformation and diagenetic processes that considerably affected the general shape of the skull and the bone surface, with several small cracks. The condition of the specimens does not permit a clear recognition of cranial sutures and also this has strongly hampered their mechanical preparation. Both specimens are quite similar in size but due to deformation comparative measurements were not taken.

The skull roof of the holotype (MCT-1716-R) was lying on the dicynodont femur and during its removal, several years ago, part of it was strongly damaged. There are still femur remains on this portion of the skull. MCT-1716-R preserves most of the right lateral aspect of the skull, including part of the snout, zygomatic arch, and a shifted squamosal portion (Figs 3 and 4). The primary and secondary palates and part of the basicranial region are partially preserved, being the latter portion shifted downward and anteriorly (Figs 4 and 5). Both lower jaws are articulated to the skull and preserve most of the postdentary elements (Figs 3 and 6). The upper dentition is well-preserved in this specimen (Figs 7 and 8) whereas the lower one is limited to two left postcanines (Fig 8C).

The referred specimen MCT-1716-R is strongly concreted and deformed (Figs 9 and 10). Overall, only the right lateral aspect of the skull and the left aspect of the snout and lower jaw can be properly discerned (Figs 9 and 10). As observed in the right lateral view, the snout and jaws are bent to the right side. The dentition is relatively well preserved in this specimen, with some partial incisors, both upper canines and left partial upper and lower postcanines (Fig 11). Based on this specimen, we can estimate that the antorbital portion of the skull is shorter than the temporal portion.

#### Skull

Fragments of premaxilla are observed on the right side of the holotype and left side of MCT-1717-R, but its shape is indiscernible. The premaxilla-maxilla suture starts just posterior to the I4. It goes dorsally and then posterodorsal. The limits of the external nares and suture with the septomaxilla are not evident. A portion of the dorsomedial process of the maxilla is observed in the holotype. According to the placement of incisors and canine, there are diastemata between I3 and I4 and between I4 and canine. The canine in the holotype is not fully erupted; therefore it would be positioned slightly posterior than its functional position, thereby reducing the canine-I4 diastema.

The maxilla is best preserved in the right side of the holotype (Fig 3). The facial process of the maxilla is dorsoventrally tall and anteroposteriorly concave, with the deepest region at the level of the fourth and fifth postcanines.

There are at least three large infraorbital foramina on the right maxilla in the holotype. The largest one is above PC2-PC3, whereas the others are above the canine and at the premaxilla-maxilla suture. In MCT-1717-R, there are at least two small foramina at the level of canine and PC1. A large depression/foramen, perhaps amplified by deformation, above PC1-PC2 could be also indicating one infraorbital foramen (Fig 10). Frequently, basal forms such as *Probainognathus*, *Chiniquodon* and *Therioherpeton* [16, 40, 41, 100] have multiple exits of the infraorbital canal. The lack of more other exits in *Bonacynodon* would be likely a preservation artifact.

The alveolar border of the snout is slightly sigmoidal in lateral view, with the lowest point at the level of the PC5. The typical angulation of chiniquodontids (e.g., [15]), between the ventral edge of the maxillary zygomatic process and the anteroventral margin of the jugal, is not evident in *Bonacynodon*. In ventral view, the axis of the posterior part of the postcanine row projects medial to the subtemporal fenestra, with the last postcanine positioned at the level of the anterior rim of the orbit. There is no a maxillary platform lateral to the tooth row.

The anteroventral border of the orbit is preserved in both specimens, but the contact and/or contribution of jugal and lacrimal are unknown due to the lack of discernable sutures. In the holotype, below the anterior orbital edge, the maxilla overlaps the jugal, posterior to the last postcanine (PC6), and only a short suture is observed (Fig 3). At this point, the jugal seems to be dorsoventrally expanded and just posteriorly it becomes low to form the lateroventral edge of the orbit, where it has its lowest height (Fig 3). Its postorbital process is well defined, flat and projects dorsally. The posterior half of the jugal is also dorsoventrally expanded and has an acute triangular surface to accommodate the anterior projection of the squamosal. On this facet, there is a small piece of bone that likely corresponds to a squamosal fragment (Figs 3 and 4).

Part of the squamosal body is shifted from its original position (Figs 3 and 4). It preserves the temporal face of the lambdoidal crest, but its zygomatic process is broken off. The antero-lateral border of the lambdoidal crest has a rugouse texture for muscle attachment. Medial to the squamosal, there is a sub-rectangular bone that should be the tabular bone. This portion is badly preserved. A post-temporal foramen is not observed. The overall aspect of the temporal portion of the skull is observed in MCT-1717-R. The parietal crest is low and the posterior portion of the zygomatic arch seems to be relatively low, for example, in comparison to *Probainognathus* [63]. In the referred specimen, the lambdoidal crest and the zygomatic arch are separated by a deep, V-shaped notch.

The palate is poorly preserved in the holotype (Fig 4). Only the left palatal processes of the maxilla and palatine are observed. They are partially separated due to deformation and incomplete preservation. Only the posterior portion of the right side of the secondary palate is evident. It does not extend until the level of the last postcanine but rather ends slightly anterior to it, as is observed in *Probainognathus* (MCZ 4279; PVL 4673; [63]). A small portion of the left palatine wedges into the pterygoid, forming the laterodorsal edge of the choana. Most of the body of the pterygoid is observed in the holotype, being transversely wide. The pterygoid wings are not fully preserved, with only a small piece of the most ventral tip that is attached to the right dentary. Posterior to the median pterygoid crest, the basicranial region is strongly deformed, being anteroventrally shifted.

Fig 5 shows our interpretation of the badly preserved basicranial region of the holotype. The occipital condyles are quite large and transversely elongated. On the right side, there is a clear fossa (i.e., jugular fossa) anterolateral to the condyle, with at least two hypoglossal foramina (condylar foramina) on the posterior wall, as in *Probainognathus* [101]. The fenestra ovalis is positioned anterior to the jugular fossa. It is relatively large and a clear rim cannot be observed. Lateral to the jugular fossa, there is a conspicuous process that could correspond to the paroccipital process, but it is shifted from its original position. The basisphenoid and basioccipital are partially preserved, with the right anterolateral portion of basisphenoid surface eroded. Anteromedially to the fenestra ovalis, there are two small foramina that could correspond to the primary facial foramen and prootic canal [101]. The left cavum epiptericum is limited by the basisphenoid and a lamina of bone, which should correspond to the quadrate ramus of the epipterygoid. A portion of prootic/opistotic is apparently preserved on the right side.

#### Lower jaws

**Fig 13:**
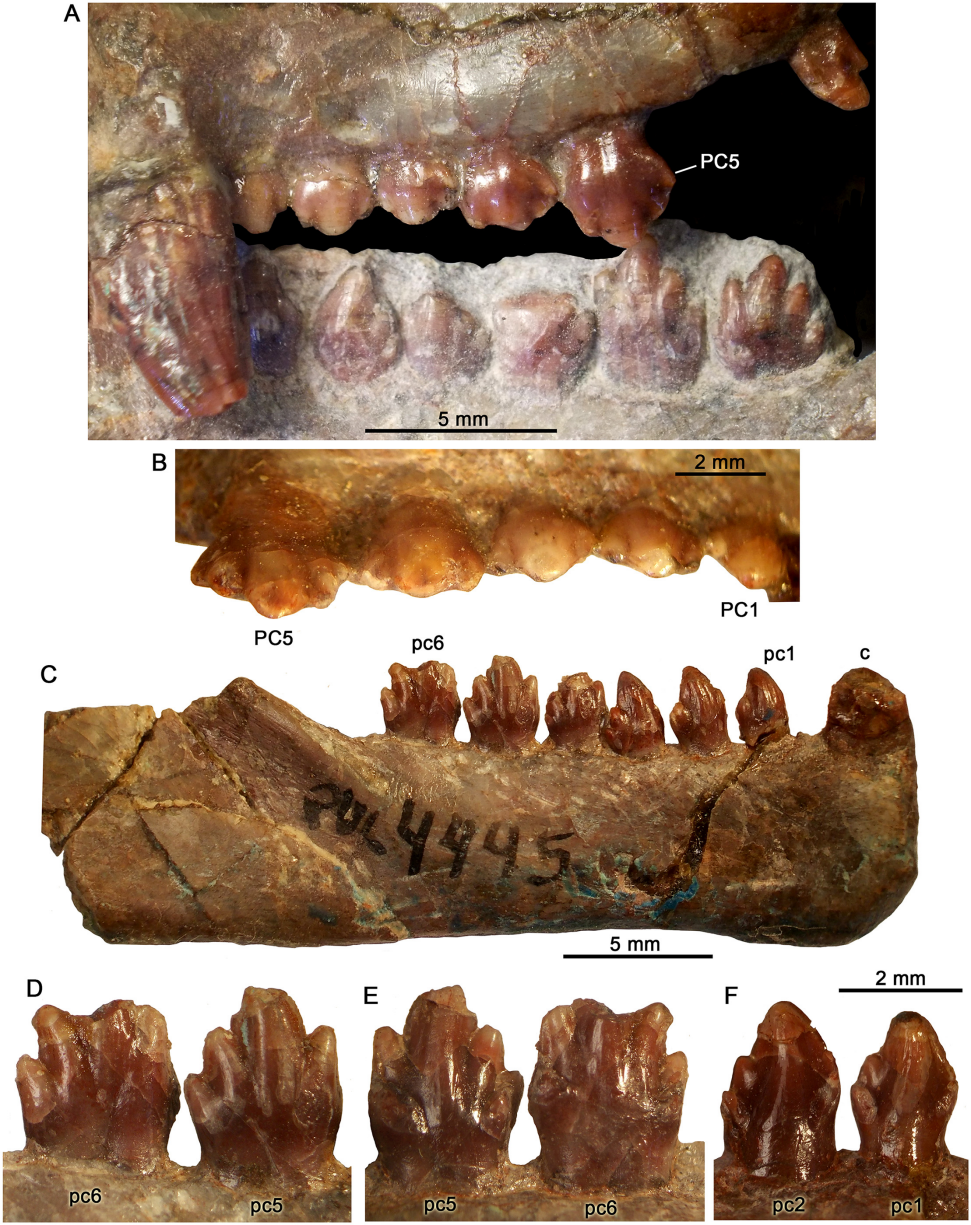
Details of the postcanine dentition of *Probainognathus jenseni* from Chañares Formation, Ischigualasto-Villa Unión Basin, western Argentina. Upper and lower postcanines (A) and detail of the lingual aspect of the upper postcanines (B) (picture taken in ventrolingual view) of PVL 4673. Right lower jaw in labial view (C) and detail of pc5 and pc6 in labial (D) and lingual (E) views of PVL 4445. Left lower pc1and pc2 in lingual view (F) of PVL 4446.

The dentary bone is preserved in both specimens whereas the postdentary complex is better preserved in the holotype (Figs 4 and 6). The dentary is moderately high in comparison to *Brasilodon* and *Brasilitherium*, but of similar relative height to *Probainognathus* (PVL 4445, PVL 4446; Fig 13) and several other probainognathians (e.g., *Chiniquodon*, PVL 4444; *Prozostrodon*, UFRGS-PV-0248-T), and slightly dorsoventrally convex in the region of the postcanine teeth. Its symphysial portion is slightly upwardly projecting and the symphysial suture is long, extended posteriorly to the level of the upper canine (due to the jaw is closed). The symphysial area has a small anteroventrally projected surface with some foramina. In MCT-1716-R, the right lower jaw is shifted from its counterpart and this likely indicates that the symphysis was unfused (Figs 3 and 4), contrary to what is observed in *Probainognathus* (PVL 4169, PVL 4678) and many other epicynodonts [63]. In MCT-1717-R the jaws are medially collapsed, with the symphysis opened. Therefore, both cases are indicating that the symphysis is unfused in this taxon. The horizontal rami of the dentary diverge posteriorly after the symphysis, forming an acute V-shaped angle in ventral view. The ventral edge of the horizontal ramus is slightly concave in lateral view and transversely broad along the level of the tooth row region. Just posterior to it, the ventral edge narrows and turns posterodorsally to form part of the postdentary trough. There is no distinct angular process. At least three mental foramina are observed in the right jaw of the holotype, two small positioned at mid-way of the symphysis, below the level of canine-I4 diastema. The large mental foramen is positioned posterior to the posterior level of the symphysis, below the level of PC2 (Fig 3).

The coronoid process is tall and posterodorsally projected. The anterior coronoid crest is thick, forming an angle of approximately 140° with the alveolar border of the dentary in MCT-1716-R and MCT-1717-R. The dorsal edge of the process is slightly convex and the posterior one is concave. The masseteric fossa develops on the entire lateral surface of the coronoid process and extends anteriorly to the level of the last postcanine. The articular (i.e., condylar) process extends far posteriorly, to reach the level of the posterodorsal corner of the coronoid process. In medial view, the Meckelian groove and postdentary trough is visible on the left lower jaw of the holotype. The Meckelian groove seems large and parallels the ventral edge of the horizontal ramus (Fig 3).

The postdentary bone complex (i.e., angular, surangular, prearticular, and articular) are observed on both sides in the holotype, with the left one dorsally shifted from its original position (Figs 3 and 6). The postdentary complex forms a reduced rod-like bone, in which discrete bones cannot be distinguished (Fig 6). The size of this bone complex is similar to that of other basal probainognathians [84, 102–104], slightly larger than in advanced non-mammaliaform cynodonts (e.g., *Brasilitherium*, UFRGS-PV-1043-T; *Riograndia*, UFRGS-PV-0596-T). The surangular is visible in medial view (Fig 6). It is short and forms a sharp dorsal crest. This crest extends anteriorly to form the upper edge of the articular+prearticular bones. Both the dorsal crest of the articular+prearticular and the angular delimit a concave area (Fig 6). A small portion of reflected lamina is preserved. It is a thin, curved process, smaller than in *Thrinaxodon* and *Lumkuia* [63, 103]. A flat splenial bone is observed in both specimens. This bone does not reach the ventral edge of the dentary and extend anteriorly to reach the symphysis.

#### Upper dentition

The holotype MCT-1716-R has at least four incisors preserved in the right side and a partial left (I1) (Figs 7 and 8). The space between I4 and canine is quite large in the right side, leaving a large diastema. The presence of a fifth incisor would not be totally discarded but the lack of an alveolus for it does not support it. The labial surface of the incisors is broken off, thus only the contour and internal structure are visible. They are dorsoventrally long and mesiodistally short, with circular cross-section, and subparallel mesial and distal edges that converge into a rounded apex. The incisors seem to be straight and ventrally projected. I1 to I3 are equal in size, with I4 slightly smaller, although it is badly preserved. I1 to I3 are separated among them by mean of a short space, similar to the width of an incisor (Fig 8B). I3 and I4 are even more separated, considering this space a small diastema. According to the relative position of the apex of the incisors, I1 and I3 have the crown larger than I2, possibly indicating an alternate incisor replacement, with I2 corresponding to a younger wave than the remaining ones. Due to the broken apex of I4 it is not possible to evaluate to which wave it belongs. In MCT-1716-R only a partial right incisor and the broken base of left incisors I2–I4 is observed, and they do not provide additional information.

The upper canine is the most conspicuous tooth in *Bonacynodon* (Figs 7–12). The right canine is preserved in MCT-1716-R and the right and left in MCT-1717-R. The right canine of MCT-1716-R is not fully erupted and its labial surface is partially preserved (Fig 12A). In MCT-1717-R both canines are fully erupted and also part of the labial surface is broken (Fig 12B). In both specimens there is a small diastema between the canine and I4. The canine in *Bonacynodon* is large, transversely narrow and mesiodistally elongated at the base. The labial surface is almost flat in MCT-1716-R and gently convex in MCT-1717-R. The mesial and distal edges are subtly convex and converge to the sharp apex. The mesial edge seems to have no denticles; however, this edge is poorly preserved in both specimens. There is a small wrinkle in the enamel of MCT-1716-R, near the alveolar edge level, that suggests a broken denticle on the mesial edge, but it is difficult to be confident. On the other hand, the distal edge has conspicuous, discrete denticles (Figs 9, 11 and 12). The denticles are not single folds or denticulations of the enamel layer, but instead they are constituted by dentine and the enamel cover [105]. There are at least eight denticles in the right canine of MCT-1716-R, seven in the left and 11 in the right canine of MCT-1717-R. In both specimens the density is three denticles per millimeter. In the right canine MCT-1717-R the denticles exhibit the most irregular size gradient. They decrease in size toward the base of the crown with some small denticles positioned between larger ones. Along the distal edge the denticles are triangular-shaped at mid-height and the apex, and become rectangular-shaped towards the root. The triangular denticles are asymmetrical with a long dorsal edge, strongly concave, and a short ventral edge, slightly convex, being the tip posteroventrally projected. The labial surface of the denticles is dorsoventrally convex and the contact between denticles forms a sharp cutting notch by the enamel layer. The denticles near the base of the crown are only seen in right canine MCT-1717-R (Fig 9); they are rectangular, not as protruding from the crown as the triangular ones, and the enamel layer at this region is very thin.

The upper postcanines are visible in the right maxilla of holotype MCT-1716-R (Fig 7) and left maxilla of MCT-1717-R (Fig 11); both specimens have six postcanines. Most postcanines have small cracks but the overall morphology is clearly preserved. The basic plan of the upper postcanines of *Bonacynodon* consists of an asymmetrical sectorial tooth, transversely narrow with a main cusp (A) and one mesial (B) and two distal (C, D) aligned accessory cusps, without labial cingulum. The height relationship among cusps is A>C≥B>D. The teeth are not in contact with each other, with the spacing between teeth longer in MCT-1717-R (Fig 11) than in the holotype (Fig 7).

In MCT-1716-R, PC1 is the smallest of the row, with a main cusp A and accessory cusp C and D. At this tooth the mesial edge of the cusp A is considerably longer than the distal edge and a mesial cusp B is not evident. PC1 is close to the canine, without a diastema. The crown of PC2 is badly preserved and conspicuously larger than PC1. Based on its size and contours, it is more similar to the posterior postcanines than to PC1. PC3 has also a broken crown and is not fully erupted. PC4 and PC5 are similar in size and morphology. They have a main cusp A located slightly mesial to the center of the crown with the mesial edge longer than the distal one. The main cusp is slightly convex and in PC5 there is a faint ridge that goes from the tip of the cusp until it disappears in the mid-high of the crown. The cusp B is partially preserved in PC4 and evident in PC5. The cusp B is almost at the same height than cusp C, but the former is slightly small mesiodistally. The mesial edge of cusp B is continuous with the rest of the crown forming a straight edge. In fully erupted postcanines, as is the case of PC4 and PC5, the mesial edge of the crown is long, positioning the cusp B well-separated from the alveolar margin. The cusp C is acute in PC4, with the mesial edge longer than the distal one. The contact of cusp C and A is notched. The cusp D is the smallest cusp of the crown, sub-conical with a continuous distal edge with the crown. The apex of this cusp is slightly posteroventrally projected in comparison to the remaining cusps; it is also the cusp closest to the alveolar margin. The last postcanine, PC6, is not fully erupted and the crown is only partially preserved. To note, this postcanine has a similar pattern than PC2-PC5 but considerably smaller, quite similar in size to PC1, which has the simplest morphology. The crown of PC6 is anteroventrally positioned, suggesting that during eruption it rotates to be positioned more vertically. In PC6 the cusp A, B and C are evident, but a cusp D is not seen as it is exposed.

The six upper postcanines of MCT-1717-R are almost similar to MCT-1716-R. The main difference is that PC4 is larger than PC5 (being sub-equal in MCT-1716-R) and due to better preservation, the cusps are sharper. The alveolar edge of MCT-1717-R is partially broken but it seems evident that PC1 and perhaps PC3 are not fully erupted. PC6 is also in eruption as in the holotype, but the tooth is extremely broken.

Complete roots are not seen in any specimen but according to what is observed at the crown/root contact, the root is simple and not constricted. Notably, wear facets on the cusps (by food consumption or occlusion) are not observed in any of the specimens.

#### Lower dentition

The lower incisors and canines are not available in the specimens, due to preservation and occlusion of the lower jaws. In the left dentary of the holotype, the root of the canine is partially preserved. It seems to be transversely narrow and of considerable size, as is the upper canine.

The holotype only preserves two left anterior postcanines, the broken crown of pc1 and a complete pc2, which are only available in labial view (Fig 8C). The pc1 is slightly smaller than pc2. The morphology of pc1 is not clear. The pc2 has a dorsoventrally tall and mesiodistally short crown, with a main cusp a and accessory cusps b, c, and d (Fig 8C). The height relationship among cusps in this anterior postcanine is a>c>b>d. The cusp a is tall, positioned in the mesial haft of the crown, with the mesial edge more convex and longer than the distal one. The tip of the cusp a is directly apically, as in the remaining cusps. Cusp b is smaller than cusp c. Cusp c has a short mesial edge and a long and straight distal margin that descent to the cusp d. Cusp d is the smallest, slightly posterodorsally projected. The mesial and distal edges of the crown taper slightly to the root, but there is not a distinctive neck. The root is barely visible and does not exhibit a constriction.

In MCT-1717-R, three left lower postcanines are partially preserved (Fig 11). They possible correspond to pc3 to pc5, according to the relative position of the closed lower jaw and the upper canine. These postcanines increase in size posteriorly, and the last one seems to be incompletely erupted. The crown of pc3 is totally crushed and the pc4 is only partially preserved. In pc4 the mesial portion of the crown where the cusp b should be placed is covered by the anterior tooth. The main large cusp a of pc 4 is followed by cusp c and d. The relative size of pc3-pc4 is considerably larger than that of pc2 of the holotype specimen. Based on the two specimens, it is possible that the anterior postcanines (pc1-pc2) are considerably smaller than the remaining ones, the same pattern that occur in the upper tooth row. The last postcanine has a well-preserved crown (Fig 11). The pc5 is transversely narrow, without evidence of cingulum with four aligned cusps. To note, the size and position of the cusp b in the pc5 of MCT-1717-R is quite different to that of pc2 of the holotype. In the pc5 of MCT-1717-R, the cusp b is in a higher position than cusp c and both have a similar size. As such, the height relationship among cusps in pc5 is a>b>c>d. According to this trait, cusp a is relatively small in comparison to that of pc2, with short and similar-length mesial and distal edges. The cusp b is in a high position in pc5. The cusp c remains lower to cusp b, with a short mesial edge and a long distal one. The cusp d is small and well-separated, basally from the remaining cusps. The notch between cusps a/c is deeper than between cusps b/a. The last postcanine is positioned just anterior to the base of the coronoid process.

#### Tooth replacement

Based on the two available specimens, tooth replacement in *Bonacynodon* is difficult to access. A typical alternate replacement as in basal non-gomphodont cynodonts [106, 107] is likely evident in the upper incisors of the holotype but regarding to the postcanine row there is little information. In both specimens most postcanines are apparently functional (perhaps, the left last postcanine of MCT-1717-R is not fully erupted) with the last upper tooth apparently in process of eruption. In addition, the preserved cusps of the postcanines are unworn. In the close relative *Probainognathus*, Romer [41] commented on the presence of heavy wear on the postcanines of old individuals, producing a chisel-like distal shearing edge. This would be indicating a reduction of the dental replacement frequency in *Probainognathus* [108]. Also, *Bonacynodon* and *Probainognathus* share the presence of a last upper postcanine reduced in size. According to Abdala et al. [107], this size variation is more pronounced in juveniles and subadults than in adult individuals of *Probainognathus*. Based on the evidence at hand of *Bonacynodon*, both individuals could be considered subadults and tooth wear is expected in more advanced stages, if a reduction in tooth replacement frequency exists.

### Comparisons

The main differences observed in *Bonacynodon* with regard to other probainognathian cynodonts are in shape of the canine and postcanine dentition. These differ from the contemporaneous *Probainognathus jenseni* from Chañares Formation by possessing a more mesiodistal lengthy upper canine with denticulated distal edge (Fig 12). In *Probainognathus* (PVL 4169, PVL 4673) the upper canine is also quite flattened transversely (Fig 12D), but denticles at the mesial and distal edges were never observed in this taxon. In the coeval *Chiniquodon* (PVL 4444), the upper canine is sub-conical and curved posteriorly (Fig 12E), without denticulated margins. *Protheriodon* (UFRGS-PV-0962-T) and *Candelariodon* (MMACR-PV-0001-T), also from the same AZ, lack serrated margins in the available canines (uppers in the former and lowers in the latter taxon). Conspicuous denticles in the upper canines, as those present in *Bonacynodon*, are uncommon in other small-sized probainognathians (e.g., *Lumkuia*, [63]; *Prozostrodon*, UFRGS-PV-0248-T; *Botucaraitherium*, MMACR-PV-003-T; *Brasilitherium*, UFRGS-PV-0929-T; *Riograndia*, UFRGS-PV-0596-T; *Irajatherium*, UFRGS-PV-0599-T). Among probainognathians, conspicuous serrations are only present in the labiolingually compressed canines of *Trucidocynodon* (UFRGS-PV-1051-T) (Fig 12D), *Ecteninion* [21], and *Diegocanis* [22]. However, the denticles in the upper and lower canines of ecteniniids (even in juvenile specimens) are small and delicate in comparison to those of *Bonacynodon* (Fig 12).

The upper and lower postcanines of *Bonacynodon* approach the morphology present in *Probainognathus*, more than any other probainognathian. Although *Probainognathus* has been known for over 40 years, there is little published data on its postcanines [109]. *Probainognathus* has 6 to 7 postcanines and in some specimens the unworn dentition is very well-preserved (e.g., PVL, 4673, PVL 4445 and 4446; Fig 13). The postcanines increase in size and complexity posteriorly. They are separated one to each other by a little space, precluding a mesiodistal contact between teeth of the same row. The crowns of middle and posterior upper and all lower postcanines are asymmetrical. The crowns of the upper postcanines are lower and mesiodistally longer than the lower ones, and in general aspect they are smaller than the lower ones. The anterior upper postcanines (pc1-pc2; specimen PVL 4673) are symmetrical with a main central cusp a and accessory cusps b and c of similar size. In the posterior ones teeth, the crown becomes larger with the addition of a conspicuous distal cusp d (Fig 13). The cusp d is slightly bent to the lingual side in pc4 and pc5 of PVL 4673. The lingual displacement of cusp d gives a slightly concave lingual surface, with conspicuous convex cusps. In the labial side, there is no cingulum (Fig 13). Lower postcanines have a conspicuous main cusp a placed on the anterior half of the crown. It has the labial surface more convex than the lingual one, and its mesial cutting edge is usually more convex and longer than the distal one. Curved cusps are not present. The first postcanine does not have an accessory cusp b. Cusp b appears subtly developed in pc2-3 and is conspicuous in the posterior ones. In the posterior postcanines, the cusp b is positioned slightly below or at the same level than distal cusp c. The cusp c is always present in the postcanines. It has a basal position in pc 1–3 and occupies a higher position in the remaining teeth. Posterior to cusp c is the cusp d that is well-evident in the last two postcanines. It is lower in position than cusp b and is slightly posterodorsally projected. All these cusps are mesiodistally aligned. In labial view, the base of the crown and each cusp are globular whereas in lingual view the concavity of the main cusp is centered at its midline and near to the mesial and distal edge these surfaces are slightly concave. Also, between cusps there is a conspicuous groove. Based on the latter traits, the postcanines of *Probainognathus* are not as narrow as, for example, *Rewaconodon* [110], *Pseudotriconodon* [111], *Lepagia* [112], or *Riograndia* [29]. In addition, the lower postcanines have a faint cingulum restricted to the mesiolingual and distolingual corners, which is not continuous and lacks conspicuous cusps (Fig 13). Comparing postcanines, subtle differences in the relative size of the cusps and the tall of the crowns are present between *Probainognathus* and *Bonacynodon*.

**Fig 14:**
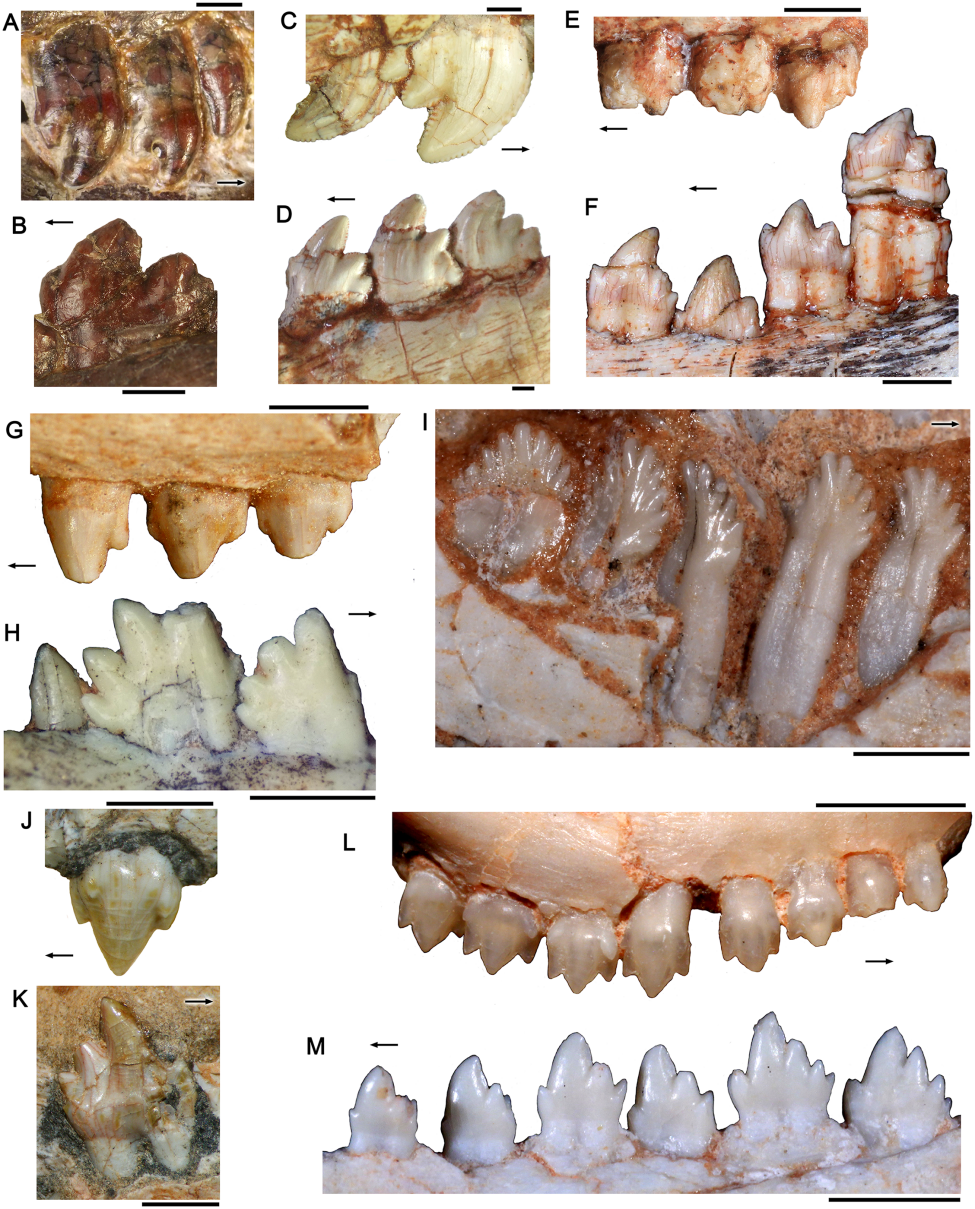
Selected postcanines of probainognathians used for comparisons. A, B, *Chiniquodon theotonicus*, anterior right upper (A) and posterior left lower (B) postcanines in labial view (PVL 4444). C, D, *Trucidocynodon riograndensis*, posterior right upper (C) and left lower postcanines in labial view (UFRGS-PV-1051-T). E, F, *Prozostrodon brasiliensis*, posterior left upper (E) and lower (F) postcanines in labial view (UFRGS-PV-0248-T). G, H, *Irajatherium hernandezi*, left upper (G, UFRGS-PV-1175-T) and posterior right lower (H, UFRGS-PV-1029-T) in labial view. I, *Riograndia guaibensis*, left lower postcanines in lingual view (UFRGS-PV-0833T). J, K, *Botucaraitherium belarminoi*, left upper postcanine in labial view (J) and posterior left postcanine in lingual view (K) (MMACR-PV-003-T). L, *Brasilodon quadrangularis*, right upper postcanines in labial view (UFRGS-PV-0611T). M, *Brasilitherium riograndensis*, left lower postcanines in labial view (UFRGS-PV-0603T). The arrows indicate mesial side. Scale bar equals 2 mm.

*Bonacynodon* differs from *Lumkuia* [63], *Mitredon* [113], *Chiniquodon* ([100]; PVL 4444; Fig 14), and ecteniniids (Fig 14) because all but the former have crowns where the main cusps are curved backwards. In addition, the margins of the postcanine cusps of ecteniniids are finely denticulated ([21], PVSJ 422; UFRGS-PV-1051-T; Fig 14). *Bonacynodon* differs from *Candelariodon*, from the same AZ, because in the latter taxon the postcanines have more bulbous cusps and there is a clear distinction between anterior and posterior postcanines (MMACR-PV-0001-T), compared to the gradual morphologic changes observed in the tooth row of *Bonacynodon*. The dentition in *Protheriodon* is badly preserved but it is quite different from that of *Bonacynodon*. Based on the middle left upper postcanines of *Protheriodon* (UFRGS-PV-0962-T), upper cheek teeth have a symmetrical and bulbous crown. The main cusp (a) is globular, with convex labial surface, and the mesial (B) and distal (C) cups are small, differing from the condition of *Bonacynodon*.

Notably, most prozostrodontians have clearly differentiated morphologies between the upper and the lower tooth rows (except for *Riograndia*), which is not seen in *Bonacynodon* or most other non-prozostrodontians cynodonts [16, 18, 19, 24] (Fig 14). The postcanine morphology of *Riograndia* and tritheledontids [24, 29, 112] is clearly different from that of *Bonacynodon* (Fig 14). There are several small cusps (up to nine) in the cutting edges of the non-cingulated, flattened upper and lower postcanines of *Riograndia* [29] (Fig 14). In tritheledontids, the upper postcanines have a transversely broad main cusp, with small mesial and distal cusps (e.g., *Irajatherium*, UFRGS-PV-0599-T; *Chaliminia*, PULR 081; *Elliotherium*, [114]), with labial cingulum in more derived forms (e.g., *Pachygenelus*, SAM 1315, 1329, 1394; [115]). *Bonacynodon* differs from *Brasilodon*, *Brasilitherium*, *Minicynodon* and *Botucaraitherium* [11, 18, 19, 31] because in the latter taxa the upper postcanines have a symmetrical pattern with a prominent central cusp (A), one mesial (B) and one distal (C) small accessory cusps aligned on the lingual side, and one mesial and one distal small cusp on the labial side (Fig 14). The lower postcanines in *Brasilodon*, *Brasilitherium* [18, 19, 58] and *Botucaraitherium* [31] are quite variable in morphology (Fig 14), but they are clearly different from *Bonacynodon* because the presence of well-differentiated anterior/middle postcanines from posterior ones, lingual cingulum with discrete cusps and 8-shaped cross-section of the root.

*Bonacynodon* also differs from *Microconodon* because in the latter the cusp arrangement of upper postcanines from the middle of the tooth row is almost symmetrical. Also, *Microconodon* has at least 8 lower postcanines with incipiently divided root [116]. The horizontal ramus of the dentary is also slender in *Microconodon*, with a less developed lateral trough for postdentary bones [116].

Several Triassic sectorial-toothed cynodonts are known from Europe based on isolated remains. The dentition of *Bonacynodon* can be distinguished from most of these forms, but comparisons are limited. For example, in the isolated postcanines referred to *Pseudotriconodon wildi*, the cusps have a symmetrical arrangement with the main cusp always dominant in labial aspect (e.g., [111, 117]). In *Tricuspes* (*T*. *tuebingensis* and *T*. *sigogneauae*) the main cusp is tall and symmetrical, with more developed cusps b and c [117]. The same conspicuously flattened crown, with incipient constriction in the root, is reported in the dromatheriid *Rewaconodon tikiensis* from the Late Triassic of India [110], differing from the postcanines of *Bonacynodon*.

The posterior extension of the secondary osseous palate has been mapped in the cynodont phylogeny and as result the long secondary palates, ending posterior to or at the level of the last postcanine, was acquired more than once [63]. *Bonacynodon* has a large secondary palate but it is not extended as far posteriorly as in *Chiniquodon* (PVL 4444), *Aleodon* [36] and most prozostrodontians [19, 29]. On the other hand, the extension of the secondary palate of *Bonacynodon* is similar to *Probainognathus*, not extended posteriorly until the last postcanine [63]. Although, the morphology of the secondary palate is not well accessed in *Bonacynodon*, the contribution of the palatine seems to be smaller than the maxilla, as occurs in *Probainognathus* [41].

**Fig 15:**
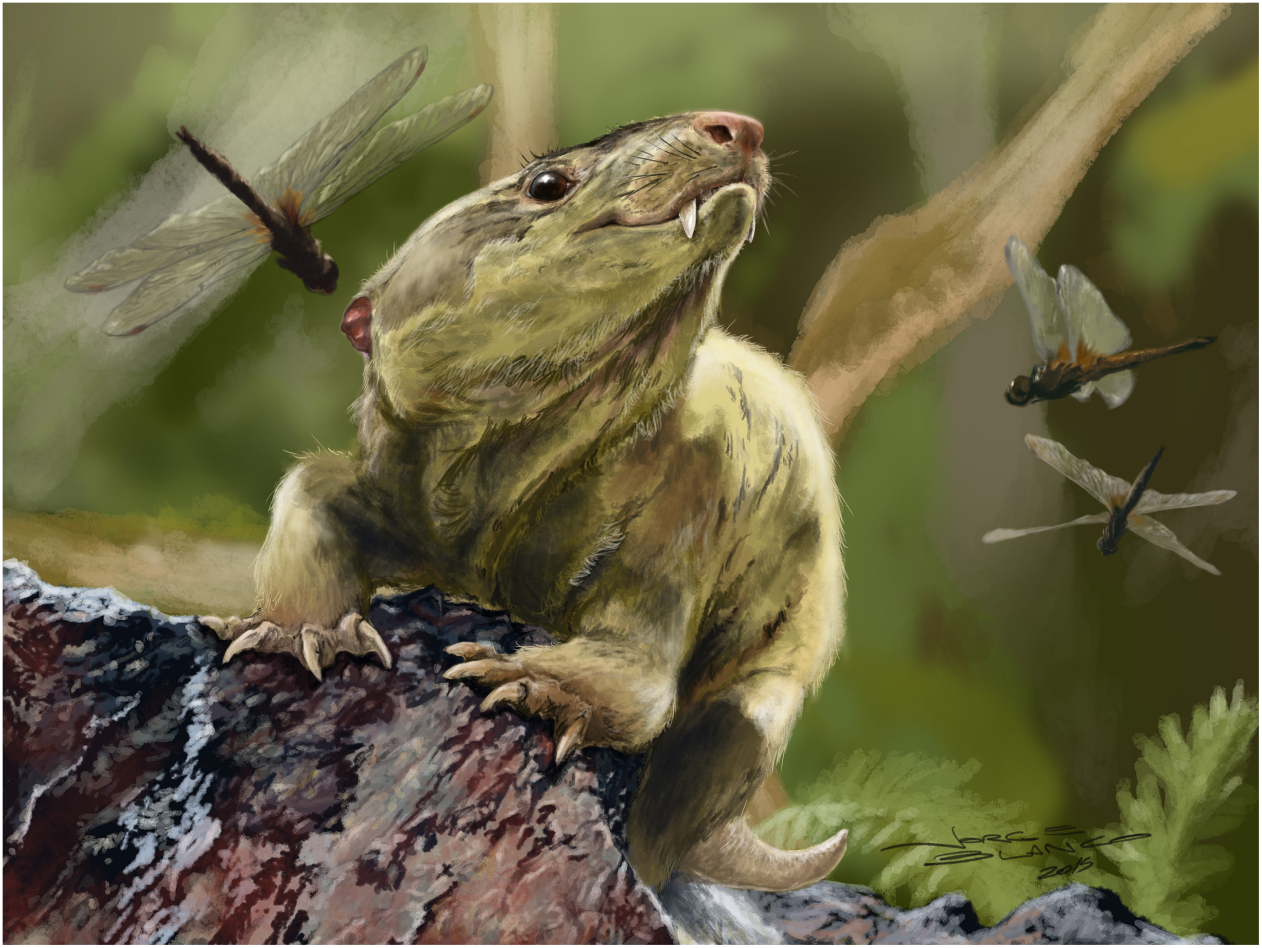
Restoration of *Bonacynodon schultzi* in life by Jorge Blanco.

The reconstruction of the lateral aspect of skull and an artistic reconstruction of *Bonacynodon schultzi* is presented in Figs 12 and 15, respectively.

### Systematic paleontology

Therapsida Broom, 1905

Cynodontia Owen, 1861

Eucynodontia Kemp, 1982

Probainognathia Hopson, 1990

*Santacruzgnathus* gen. nov.

urn:lsid:zoobank.org:act:07D885C9-8D06-4CEA-B501-8A5801678055

#### Type and only known species

*Santacruzgnathus abdalai* sp. nov.

#### Diagnosis

*Santacruzgnathus* is a small-sized cynodont with the following unique combination of features (autapomorphy with an asterisk*): low horizontal ramus of the dentary; sectorial, multicusped last postcanine, with symmetrical main cusp a in line with distal cusp c; mesial accessory cusp b labially displaced and separated from cusp a by a groove*; small distal cusp d slightly labially positioned, with an distolingual cingulum; crenulated mesiolingual cingulum; relationships of height cusps in last postcanine: a>c>b>d; incipient division of root with shallow groove only on the labial side; Meckelian groove faint, parallel to ventral edge of dentary.

#### Etymology

*Santacruz*, in reference to the municipality of Santa Cruz do Sul (state of Rio Grande do Sul, Brazil) where the holotype was found. *Gnathus* from new Latin, in reference to lower jaw and frequently used for cynodont taxa.

*Santacruzgnathus abdalai* sp. nov.

urn:lsid:zoobank.org:act:74A64197-87CC-469B-8F29-148EE0D4537C

#### Holotype

**Fig 16:**
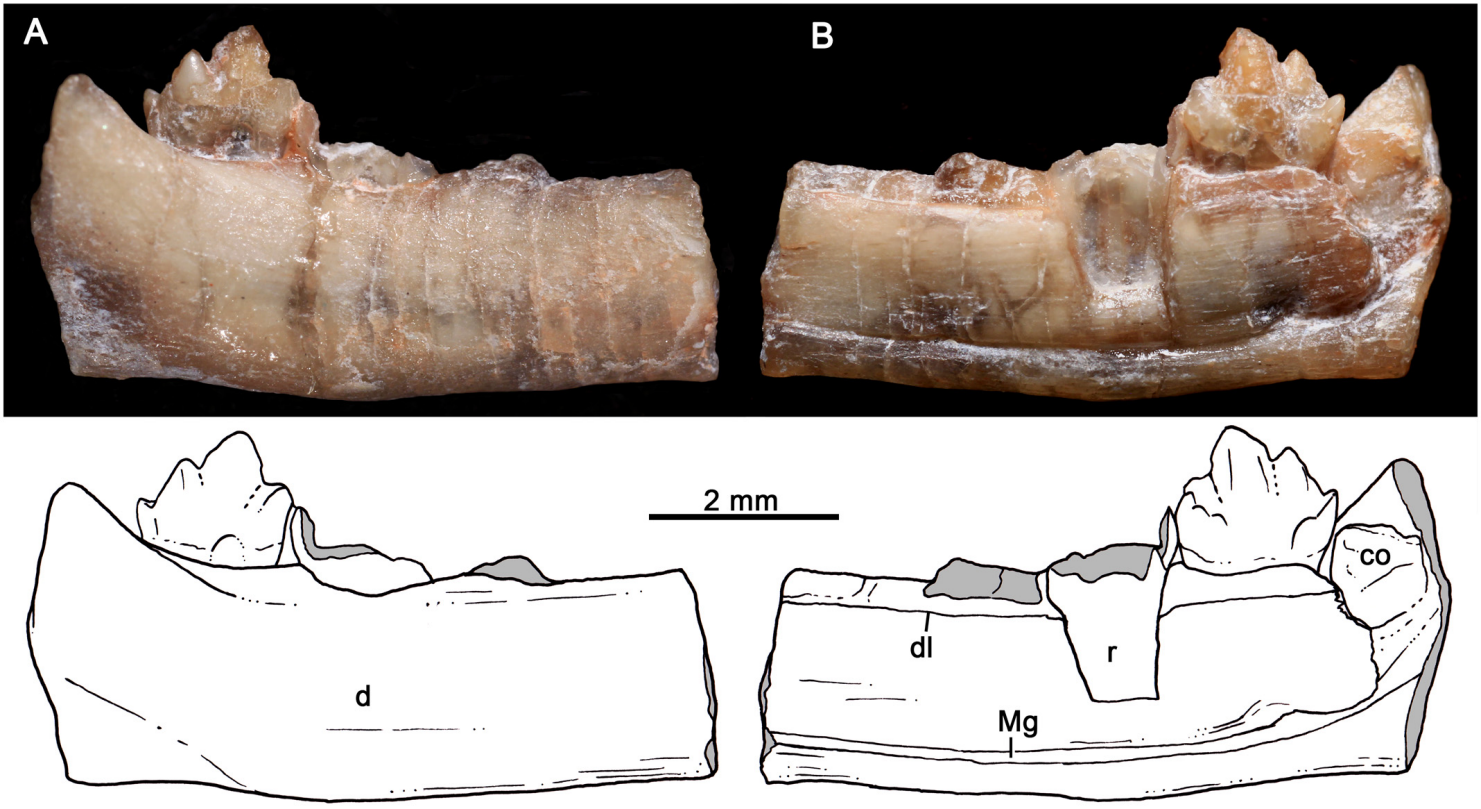
*Santacruzgnathus abdalai*, holotype UFRGS-PV-1121-T. Partial right lower jaw with complete crown of last postcanine in lateral (A) and medial (B) views. Gray indicates broken areas.

**Fig 17:**
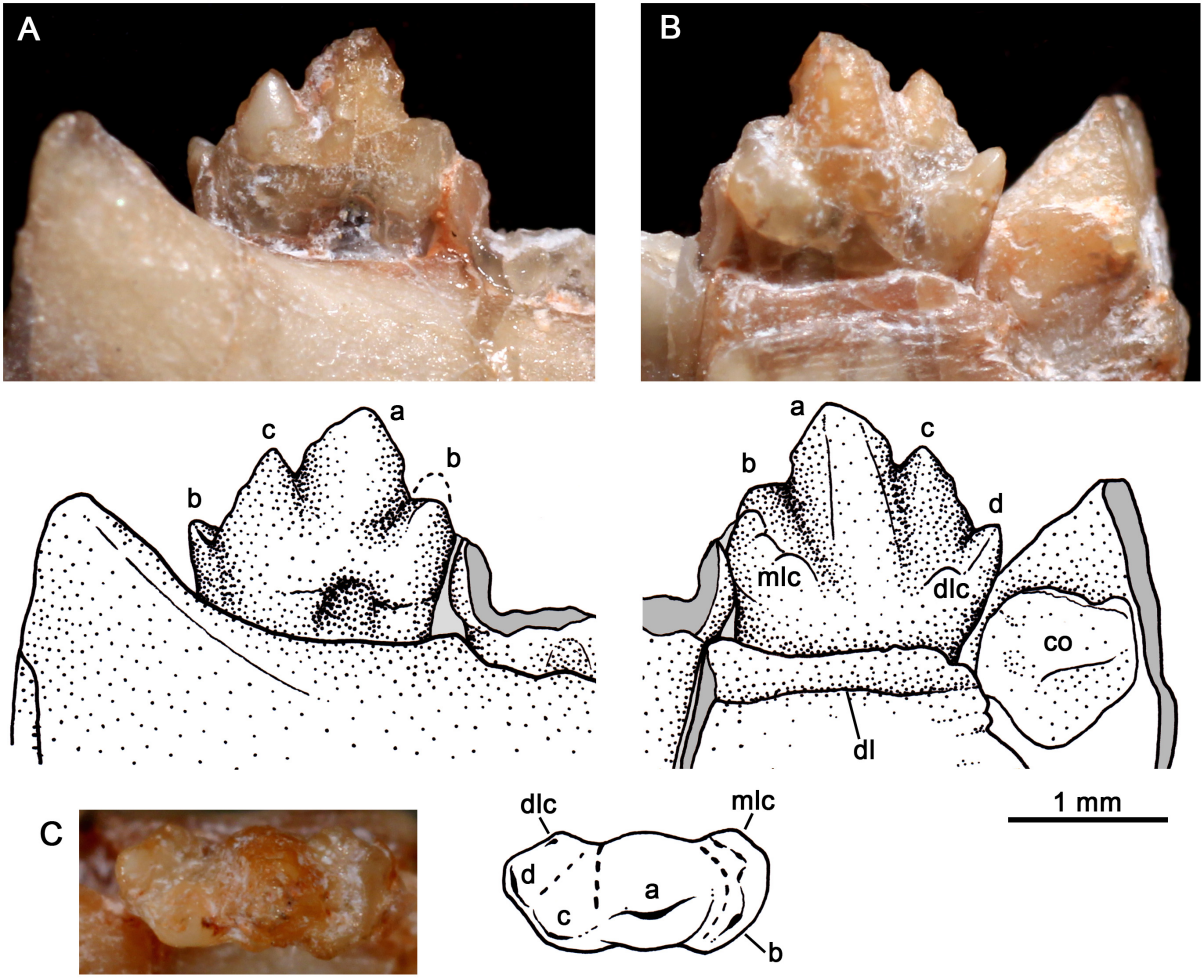
*Santacruzgnathus abdalai*, holotype UFRGS-PV-1121-T. Detail of last lower postcanine in labial (A), lingual (B) and occlusal (C) views. Gray indicates broken areas.

UFRGS-PV-1121-T, partial right lower jaw with the last postcanine (Figs 16 and 17).

#### Diagnosis

As for genus.

#### Etymology

Species named in honor to the Argentinean paleontologist Dr. Fernando Abdala, for his huge contributions in morphology, taxonomy, and phylogeny of therapsids, with emphasis on cynodonts from South America and Africa.

#### Remarks

Soares et al. [30] described this specimen as cf. *Probainognathus* from the *Santacruzodon* AZ.

#### Locality and horizon

Schoenstatt Site on federal highway BR 471, municipality of Santa Cruz do Sul, state of Rio Grande do Sul (Brazil; Fig 2). *Santacruzodon* AZ, Santa Cruz Sequence, Santa Maria Supersequence [29, 49], which is considered early Carnian in age.

#### Comments

The fossil context of the *Santacruzodon* AZ is, in comparison with other AZs from the Triassic of Brazil, significantly sparse at present. Traversodontids are the most abundant [54], with at least three recognized taxa: *Santacruzodon hopsoni* [89], *Menadon besairiei* [118], and *Massetognathus* sp. [29, 54]. Other taxa, represented by sparse material, consist of the probainognathian *Chiniquodon* sp. [54], unpublished cranial dicynodont remains [119, 120], the archosauriform *Chanaresuchus bonapartei* [119], and the pseudosuchian *Dagasuchus santacruzensis* [120]. The fossiliferous outcrops of the *Santacruzodon* AZ are quite small and geographically limited [49].

### Description and comparisons

Soares et al. [30] considered this specimen as possibly juvenile, due to its small size and direct comparisons with the wide sample of specimens of *Probainognathus jenseni* from the Argentinean Chañares Formation. However, solely the size of one specimen is not indicative of a juvenile condititon. The position of the last postcanine with regard to the base of the coronoid likely approaches the condition of subadult or adult animals. Usually, juvenile specimens have a postcanine in the process of eruption posterior to the last functional tooth and lingual to the coronoid process, as is observed, for example, in *Thrinaxodon* [106, 107]. The lack of such a posterior tooth and the functional condition of the last postcanine are not indicative of a juvenile condition of the *Santacruzgnathus* holotype.

Based on the preserved portion of the horizontal ramus of the dentary, it is slender, low, with the lateral surface dorsoventrally convex similar to prozostrodontians, such as *Brasilodon* and *Brasilitherium* [18, 19, 58]. The alveolar and the ventral margins of the dentary are straight and parallel (Fig 16). In lingual view, a faint Meckelian groove extends parallel and close to the ventral edge of the dentary. Ventral to the last postcanine, the groove slightly expands dorsoventrally at the join with the postdentary trough. Parallel to the lingual alveolar edge, a shallow groove for the dental lamina is evident. The coronoid bone, not mentioned in Soares et al. [30], is partially preserved as a thin lamina, located at the anterolingual base of the coronoid process. It is firmly attached to the dentary, and the most evident limit between both elements is observed on the dorsal portion of the coronoid bone (Fig 17). Posterior to this bone, the dentary is broken off. Only the base of the coronoid process is preserved, forming an angle of about 140° with the alveolar margin.

Anterior to the last postcanine there are broken and badly preserved roots of three postcanines that, according to the mesiodistal length of the alveoli, increased posteriorly in size. The base of the crown of the penultimate postcanine is partially preserved with part of a distolingual cusp that rests mesial and below the cusp b of the ultimate postcanine (Fig 17).

The last postcanine of *Santacruzgnathus* is distinctive from other probainognathians, especially from those cynodonts from the *Dinodontosaurus* and *Santacruzodon* AZs. The main cusp a is symmetrical, and not curved posteriorly. In line with cusp a is the distal cusp c. It has a sharp apex and its distal cutting edge is longer that its mesial one, because of the low position of cusp d. In contrast to most other basal probainognathians (*Lumkuia*, [63]; ecteniniids [21], PVSJ 422, UFRGS-PV-1051-T; chiniquodontids, PVL 4444; Fig 14), cusp b is slightly displaced lingually, separated from cusp a by an oblique groove. Because of this, cusp b and the mesiolingual cingulum remain separated from the main cusp. The mesiolingual cingulum has at least three crenulations that do not represent real cusps; it descends distolingually to merge the crown. The displacement of cusp b and the lingual cingulum produce a transversely wide mesial margin of the crown. We are not confident if it is concave to receive the cusp d of the preceding tooth (i.e., forming an interlocking mechanism), but at least the penultimate and ultimate postcanines are closely positioned one to another. The distal cusp d is sharp and posterodorsally protruding. Ventrally and lingually to it, there is a faint cingulum that does not form a real cusp. The relationship of height cusps in last postcanine is as follow: a>c>b>d. The root of this postcanine has the start of an incipient groove on its labial side. On the lingual side, there is no constriction on the root.

Importantly, some regions of the enamel of the last postcanines have suffered acid action due to the abraded condition of the enamel layer and the presence of very small pits, for example, at the posterolingual wall of the tooth. Due to their distribution on the crown, these marks are unlikely result of occlusion movements. They would be product of diagenetic process or of acid action of a predator. Nonetheless, because no marks of acid can be recognized in the dentary bone, this latter hypothesis is weak and difficult to prove with the specimen at hand.

Soares et al. [30] noted the similarities of the postcanine of *Santacruzgnathus* with young individuals of *Probainognathus* from Chañares Formation and with the specimen PVSJ 410 described as cf. *Probainognathus* from the Ischigualasto Formation [42]. They also mentioned differences with *Probainognathus*, noting that in the Argentinian taxon the postcanine crowns are relatively dorsoventrally higher and mesiodistally shorter, and the dentary is stouter than in *Santacruzgnathus*. The robustness of the dentary bone is evident in juveniles and adults of *Probainognathus* (Fig 13) and this is not the case for the lower jaw of *Santacruzgnathus*. Also, the ventral edge of the horizontal ramus of *Probainognathus* is slightly convex in lateral view (Fig 13), differing from the condition of *Santacruzgnathus*.

Our comparisons suggest that UFRGS-PV-1121-T constitutes a new probainognathian cynodont, distinct from *Probainognathus* and other cynodonts. The lack of curved cusp in the last postcanine differentiates *Santacruzgnathus* from *Lumkuia* [63], *Chiniquodon* (PVL 4444), ecteniniids (PVSJ 422; UFRGS-PV-1051-T) (Fig 14), and *Mitredon* [113]. *Candelariodon*, from the older *Dinodontosaurus* AZ, has more robust dentary [27]. The postcanine morphology of *Santacruzgnathus* likely approaches more derived probainognathians such as *Prozostrodon* [16] from the *Hyperodapedon* AZ and *Brasilodon*, *Brasilitherium* and *Batucaraitherium* [18, 19, 31, 58] from the *Riograndia* AZ (Fig 14). The complexity of the crown in these forms is conspicuous due to the presence of cingula and displacement of main cusps from a lineal, mesiodistal arrangement.

Although based on partial material, we consider *Santacruzgnathus* as a member of the Probainognathia clade due to the low and slender dentary, reduced Meckelian groove, incipient division of root, crown of last postcanine with mesiolingual and distolingual cingula, cusp b slightly lingually displaced, and closely positioned postcanines (based on penultimate and ultimate teeth). These features also indicate a close relationship of *Santacruzgnathus* with prozostrodontians rather than with basal probainognathians (*Ecteninion*, *Chiniquodon*, *Probainognathus*).

## Discussion

Our results and comparisons permit the recognition of two new cynodonts, one of which (*Bonacynodon*) was included in a cladistic analysis in order to test its phylogenetic position among cynodonts.

### Phylogenetic relationships of *Bonacynodon schultzi*

**Fig 18:**
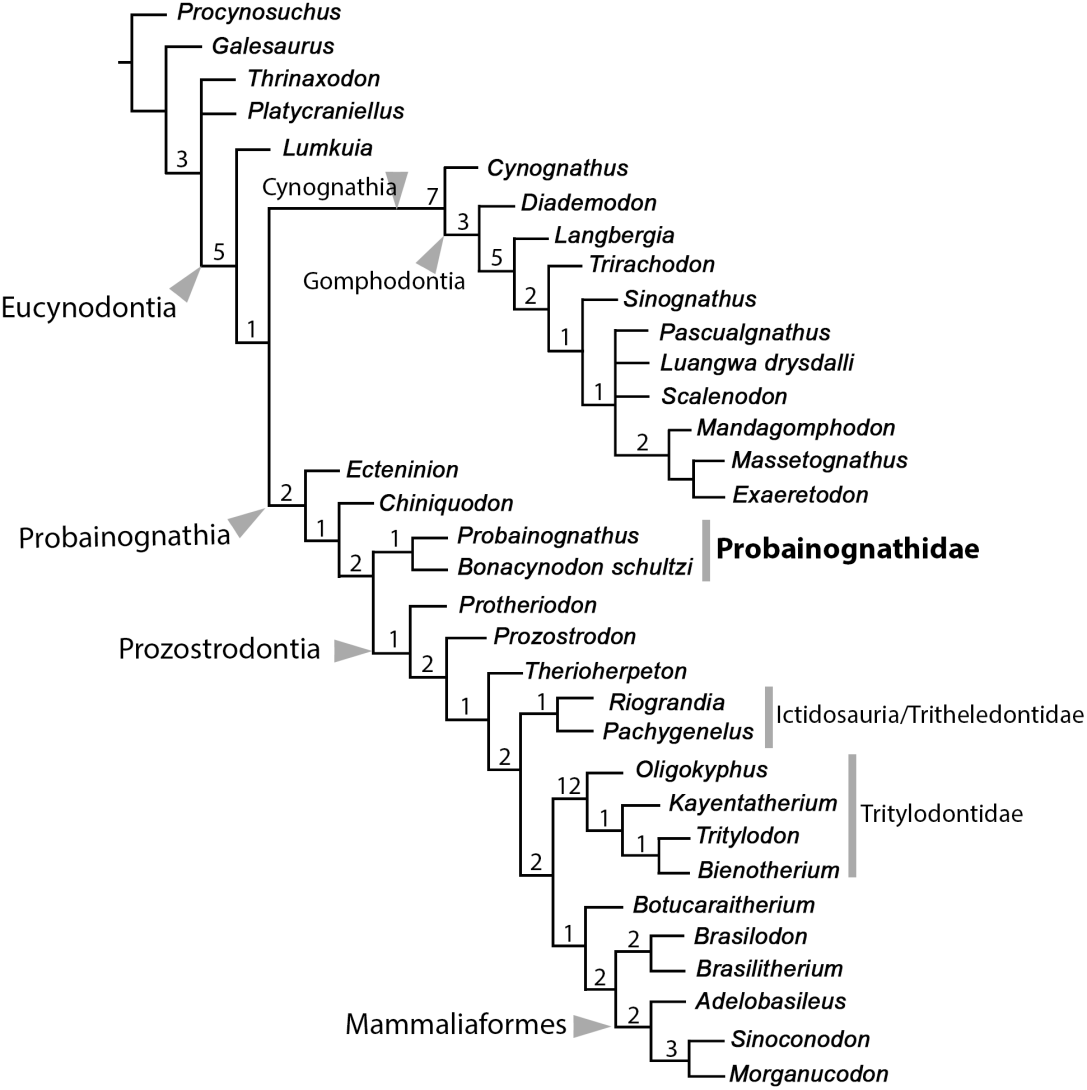
Phylogenetic analysis. Strict consensus tree (L = 450, Ci = 47, Ri = 77) of four most parsimonious trees depicting the phylogenetic position of *Bonacynodon schultzi*. Bremer support is indicated at each node.

Our analysis resulted in four most parsimonious trees of 450 steps, with a Consistency Index (CI) of 0.47 and a Retention Index (RI) of 0.78. The strict consensus tree and Bremer Index of each node are shown in Fig 18. *Bonacynodon* is positioned as sister-taxon of *Probainognathus*, together forming the family Probainognathidae (see Systematic Paleontology). This family is nested as the sister-group of *Protheriodon* plus Prozostrodontia (sensu [71]). The support of this family is low, with only one synapomorphy (Character 118, State 0): posterior end of the upper tooth row positioned below and anterior to subtemporal fenestra. Characters that support each node are listed on Supporting Information (S1 File). The position of *Lumkuia* as the basalmost Eucynodontia, sister-taxon of Cynognathia plus Probainognathia is noteworthy [71], because this taxon is traditionally placed inside the latter clade [63, 69, 90]. The modifications in character-states of *Riograndia* have affected its position in comparison to the analysis of Liu and Olsen [71], in which a *Pachygenelus* and *Riograndia* do not form a monophyletic group (i.e. Ictidosauria/Tritheledontidae). In the hypothesis here presented the clade *Riograndia* plus *Pachygenelus* is supported by the following character-states: absence of paracanine fossa (4(3)), ovoid to cylindrical occipital condyles (68(1)), and small upper canine (99(1)). In other analyses with a wide sample of Tritheledontidae and characters [24, 25], more features support this clade. The inclusion of additional South American taxa, such as *Irajatherium*, *Chaliminia*, *Candelariodon*, *Trucidocynodon*, could help in the resolution of probainognathian relationships but a larger set of characters would need to be selected, especially focusing in the tooth morphologies of the clade.

The basal species of probainognathians, such as chiniquodontids and ecteniniids, show dental features with a tendency to carnivory. They have sectorial postcanines with curved cusps that in some cases have serrated margins (see Fig 14A and 14D). In the most inclusive clade (Probainognathidae plus Prozostrodontia), the species developed multicusped crown of “triconodont type” with elaborated cingula in some forms (Figs 13 and 14) that are indicative of a more insectivorous condition. Therefore, a tendency to carnivory is apparently lost in more derived probainognathians.

### Comments on *Protheriodon*

**Fig 19:**
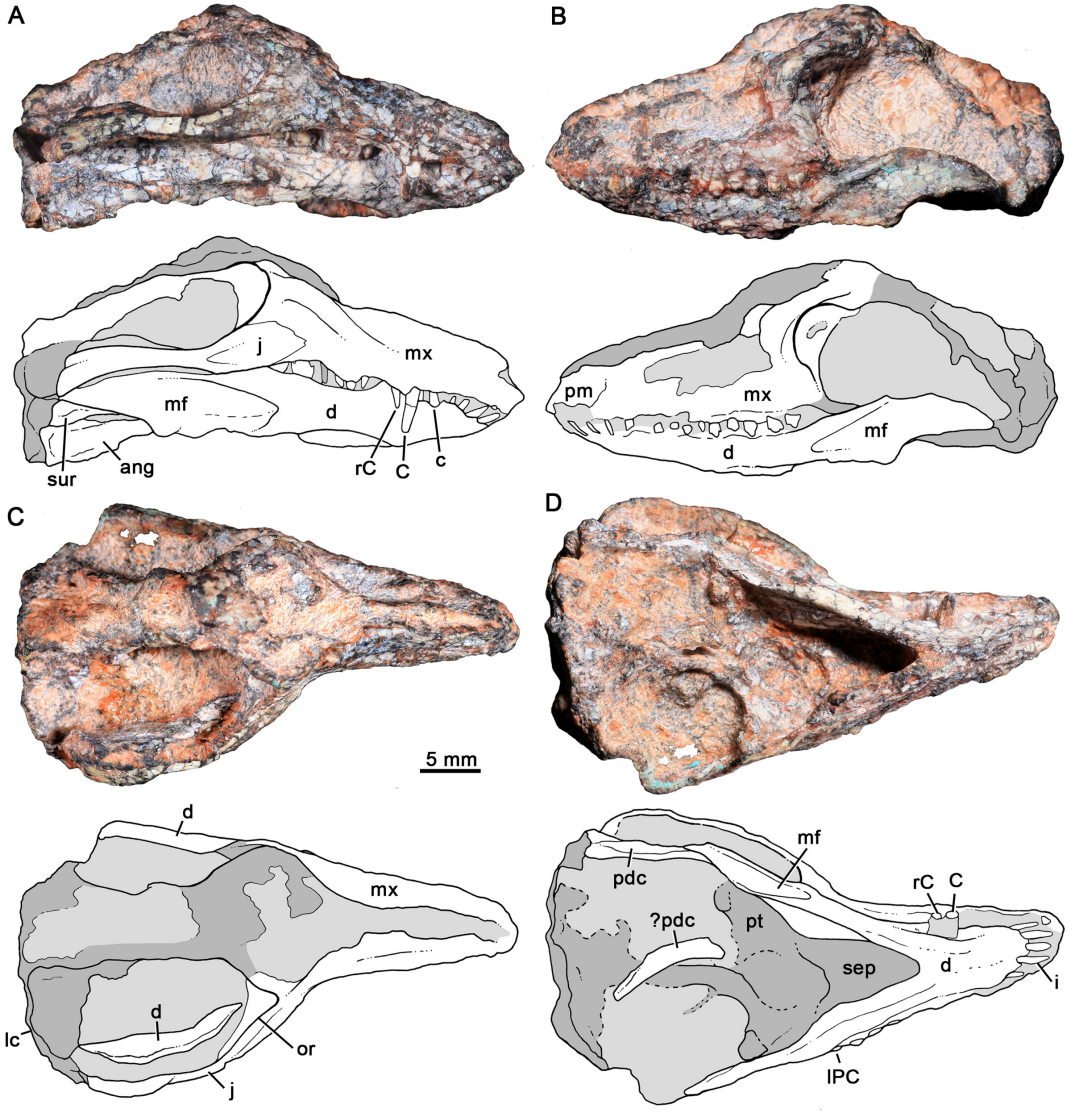
*Protheriodon estudianti* (Holotype UFRGS-PV-0962-T). Skull in right lateral (A), left lateral (B), dorsal (C), ventral (D) views. Dark grey indicates most broken areas and soft grey indicates matrix.

**Fig 20:**
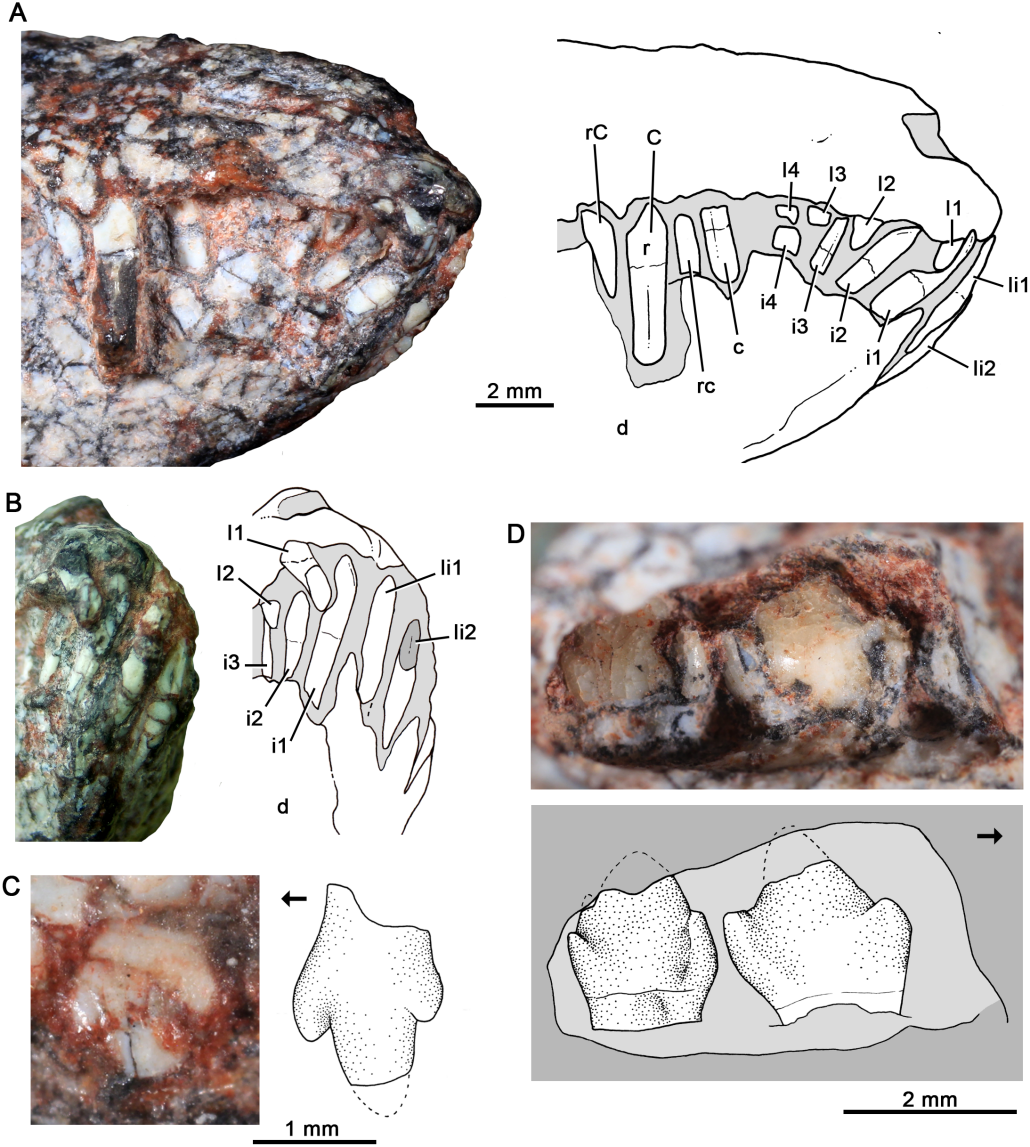
*Protheriodon estudianti* (Holotype UFRGS-PV-0962-T). Details of canines and incisors in right lateral (A) and anteroventral (B) views, middle left upper postcanine (C) and left posterior lower postcanines (D) in labial view. Dark grey indicates broken areas and soft grey indicates matrix. Arrow indicates mesial direction.

We have included *Protheriodon* in the phylogenetic analysis presented here (Fig 18). It is positioned as the sister-taxon of Prozostrodontia (*Prozostrodon* plus more inclusive taxa). The monophyly of Brasilodontidae as expressed by Bonaparte [74] is not supported here and new analyses with a larger sample of taxa and characters should be performed in order to address this problem properly. *Protheriodon estudianti* was described by Bonaparte et al. [20] based on a partial skull with articulated lower jaws. The only known specimen is the holotype UFRGS-PV-0962-T (Figs 19 and 20) found at Dona Francisca locality, state of Rio Grande do Sul (Brazil), into the *Dinodontosaurus* AZ [49] (Fig 1).

Bonaparte et al. [20] considered *Protheriodon* as a basal Brasilodontidae, with close resemblances with *Procynosuchus* from the Late Permian of Europe and Africa (e.g., [121]). In the same work, the authors proposed the presence of a lineage (sequence) within Cynodontia constituted by *Procynosuchus* + *Protheriodon* + *Brasilodon* + *Brasilitherium* + *Morganucodon* (which they termed Group 1), clearly divergent of other carnivores/insectivores taxa, including *Thrinaxodon* + *Lumkuia* + Chiniquodontidae + *Probainognathus* (termed Group 2). This dichotomy was justified mainly due to the presence of an interpterygoid vacuity, an elongated skull, and slender lower jaws in the members of the Group 1. By contrast, members of the Group 2 were thought to lack interpterygoid vacuities, the skull was axially shorter and transversely broader, and the zygomatic arch and lower jaws were more robust. In a later contribution, Bonaparte [74] added *Panchetocynodon damodariensis*, from the Early Triassic of India [122], to the Brasilodontidae group. The topology of these character-states in several phylogenetic hypotheses (e.g., [19, 26, 63, 71, 90]) did not exhibit a homogeneous (“ideal”) distribution within Cynodontia as proposed by those authors, with other features (e.g., tooth morphology, basicranial traits, etc.) also relevant in the resolution of cynodont relationships. For example, with regard to the interpterygoid vacuities, other phylogenetic hypotheses have suggested that they re-appeared within Cynodontia more than once, and in the probainognathian lineage they have an important role for remodeling the mesocranium until reach the condition present in basal mammaliaforms (e.g., *Morganucodon*; see detail discussion in [25]).

With regards to the incisors of *Protheriodon*, Bonaparte et al. [20] counted five right upper incisiform teeth plus three empty alveoli in the maxilla prior to the canine, similar to the condition (i.e., high number of incisiforms) of some specimens referred to *Brasilitherium* (UFRGS-PV-929-T, [18, 19]) and procynosuchids. Based on our observations (Fig 20A and 20B), in the right side of the holotype it is possible to see I1, I2 and two very small roots, one next to the other of I3–I4 (we are not confident if the root of I4 is actually a I4 or a replacement for I3, as occurs with the upper canine; see Fig 20A). I1, I2 and I3 are widely spaced and no discrete alveoli for other incisors are observed. The I1 is slightly larger than I2, being the crown of the latter badly preserved. The large diastema between the last preserved incisor root and the erupting canine would serve as space for an addition tooth, but there is no positive evidence for that. However, because there are apparently five lower incisors (see below), it is likely that the number of upper incisors is greater than four (but no more than five). The upper incisors seem to be relatively smaller than the lower ones. On the left side, there are no preserved incisors.

The count of lower incisors is quite difficult because it depends on the selection of the first incisor and in the fact that one side is poorly preserved. Bonaparte et al. [20] counted five lower incisors. According to our observations, there are four lower incisors on the right side, which decrease considerably in size posteriorly, with the i1 and i2 relatively large and anterodorsally projected (Fig 20A). On this side, the root of the incisors is well exposed. The left i1 and i2 are similar in size to the right homologous incisors. Our selection of left i1 as belonging to the left dentary (contrary to [20]) is justified by the presence of the anteroventral edge of the right dentary just medial to left i1 and because the left i1 is positioned lateral to the notch between the medial contact of premaxillae (Fig 20B). Consequently, *Protheriodon* has at least four (and perhaps one more) upper and four lower incisors. This incisor count is different from the condition of most eucynodonts (excluding the autapomorphic tritheledontids and tritylodontids [63, 123]), which have four upper and three lower incisors [71, 84]. More than four upper and four lower incisors is a condition present in basalmost cynodonts, such as procynosuchids (AMNH 8220; [121]), and *Prozostrodon* [16]. *Prozostrodon* has five upper and four lower incisors, also a rare condition among probainognathians. In large-sized specimens of *Brasilodon* and *Brasilitherium* there are only three lower incisors [19, 58], whereas the number of upper incisors varies considerably according to the size of the specimens. Smaller ones have more incisors (up to seven incisiviforms into premaxilla and maxilla; [19]) whereas larger specimens have only three (e.g., UFRGS-PV-1043-T; [58]). Consequently, the condition of *Protheriodon* can be interpreted as a juvenile feature, without knowing the real number of incisors in adult individuals, or a condition that is also share at least with *Prozostrodon*. The juvenile condition is also supported by the relatively large orbits [20].

As stated by Bonaparte et al. [20], the upper postcanines of *Protheriodon* (Fig 20C) have a similar morphology to *Brasilodon*, *Brasilitherium* and *Minicynodon* [11, 18, 19, 58]; however, the latter taxa have labial accessory cusps (Fig 14L). Also, the general aspect of the postcanines of *Protheriodon* is a plesiomorphic pattern within cynodonts, observed in several species such as *Procynosuchus* and *Thrinaxodon* [106, 107, 121]. The lower postcanines of *Protheriodon* are not well-preserved and the crown is only partially observed in two posterior right teeth (Fig 20D). These teeth seem to have a likely symmetrical aspect with a main conspicuous cusp a and mesial and distal cusps (Fig 20D). Apparently, the last postcanine has two distal cusps. As other probainognathians, the upper teeth are smaller than the lower ones.

Cranial sutures are difficult to observe in *Protheriodon*. The snout is long and narrow, with a slender zygomatic arch [20] (Fig 19). The lack of a postorbital bar is likely genuine but most of the skull roof, posterior to the anterodorsal rim of the orbit, is broken off (Fig 19). Although the secondary palate is long, it does not reach the last postcanines, being longer in *Prozostrodon*, *Brasilodon*, and *Brasilitherium*. Bonaparte et al. [20] mentioned the presence of an interpterygoid vacuity in *Protheriodon*. This is likely dubious, as most of the ventral aspect of the skull is crushed with a small fragment of bone glued on the primary palate. This rod of bone could be a shifted left postdentary complex. In this regard, the primary palate of *Protheriodon* likely has morphology more similar to *Probainognathus* than to *Brasilitherium*.

The presence of unfused lower jaws in *Protheriodon* (sensu [20]) is also surprising. As preserved, the lower jaws are tightly articulated, without evidence of a clear suture (Fig 19). Although the information of the holotype of *Protheriodon* is limited, it is a bizarre component of the *Dinodontosaurus* AZ (Fig 1). Its close relationship with *Brasilodon* and *Brasilitherium* as stated by Bonaparte et al. [20] is interesting although the phylogenetic hypothesis here presented positioned *Protheriodon* more basally. The mosaic of plesiomorphic and apomorphic traits seems to be the common condition among Triassic eucynodontians confounding the establishment of strong phylogenetic signals. Also, the dental diversity in probainognathians during the Middle-Upper Triassic is conspicuous; it is not reflected in the selected characters of phylogenetic analyses. Therefore, new specimens and a large database of taxa and character will better resolve cynodont relationships prior to the mammaliaform clade.

## Conclusions

We described two new cynodonts from the early Late Triassic of southern Brazil. One taxon, *Bonacynodon schultzi*, comes from the early Carnian *Dinodontosaurus* AZ. Phylogenetically, *Bonacynodon* is closer relative to *Probainognathus jenseni* than to any other probainognathian, and both are recognized as members of Probainognathidae. Among small-sized eucynodonts, *Bonacynodon* has conspicuous canines with a denticulated distal margin. The other new taxon is *Santacruzgnathus abdalai* from the *Santacruzodon* AZ. Although based solely on a partial lower jaw, it represents a probainognathian close to *Prozostrodon* from the *Hyperodapedon* AZ and *Brasilodon*, *Brasilitherium* and *Botucaraitherium* from the *Riograndia* AZ.

*Protheriodon estudianti*, from the *Dinodontosaurus* AZ, was included in the phylogenetic analysis here presented and it was nested as sister-taxon of Prozostrodontia (*Prozostrodon* plus more inclusive taxa). Some morphological aspects of its skull and dentition were re-evaluated.

The two new cynodonts and the phylogenetic hypotheses presented herein indicate the degree to which our knowledge on probainognathian cynodonts is incomplete and also the relevance of South American fossil record for understanding their evolutionary significance. The taxonomic diversity of probainognathians from Brazil and Argentina and their abundance, for example in comparison with probainognathians from North America, Europe and India (e.g., [110–113]), are and will be the base to deep and complex studies for understanding the evolutionary transformation of cynodont leading to mammals.

This is the first contribution of a project to review South American probainognathian cynodonts, and as stated along the text several issues still need to be addressed.

## Supporting Information

S1 File Phylogenetic analysis. List of changes in character definition and scorings in taxa, data matrix, and list of synapomorphies for probainognathian clades.
(DOCX)
