# Supplementary material for: Two New Cynodonts (Therapsida) from the Middle-Early Late Triassic of Brazil and Comments on South American Probainognathians
Source: PLoS One. 2016 Oct 5;11(10):e0162945. doi: 10.1371/journal.pone.0162945 (PMC5051967; doi:10.1371/journal.pone.0162945)
Supplement: S1 File — List of changes in character definition and scorings in taxa, data matrix, and list of synapomorphies for probainognathian clades. (DOCX) [file pone.0162945.s001.docx]

**Supporting Information for Martinelli et al. “Two new cynodonts (Therapsida) from the Middle-early Late Triassic of Brazil and comments on South American probainognathians”**

**APPENDIX SI**

List of changes done to the data matrix of Liu and Olsen (2010) for the current analysis. Some of these modifications were done in the analysis of Soares et al. (2014). Those modifications and several other are listed below:

**(1) Changes in character definition:**

**Character 37** [modified according to Hopson and Kitching (2001), Martinelli and Rougier (2007): ch. 47]: Length of palatine relative to maxilla in secondary palate: shorter (0); about equal (1); longer (2).

**Character 96** (redefined): Incisors: all of similar size (0); some incisor large (1).

**Character 106** (new state and re-ordered): Upper postcanine roots: single (0); constricted root, with incipient longitudinal groove (1); divided into two longitudinal aligned roots (2); multiple roots (more than two) (3).

**Character 107** (new state and re-ordered): Lower postcanine roots: single (0); constricted root, with incipient longitudinal groove (1); divided (2).

**2) Changes in scorings of taxa:**

**Character 01:** *Prozostrodon* changes from 1 to 2 (based on Bonaparte and Barberena 2001). *Riograndia* changes from 0 to 2 (based on Bonaparte et al. 2001, Soares et al. 2011).

**Character 2:** *Riograndia* changes from 1 to 0.

**Character 07:** *Therioherpeton* changes from ? to 1 (based on Bonaparte and Barberena 2001).

**Character 11:** *Brasilodon* changes from 1 to 2.

**Character 13:** *Prozostrodon* changes from 0 to 1 (based on UFRGS-PV-0248-T, Martinelli et al. 2005, Martinelli and Rougier 2007).

**Character 14:** *Prozostrodon* changes from 1 to 2 (based on UFRGS-PV-0248-T, Martinelli et al. 2005, Martinelli and Rougier 2007).

**Character 17:** *Riograndia* changes from ? to 2 (based on Bonaparte et al. 2001, Soares et al. 2011).

**Character 19:** *Riograndia* changes from ? to 0 (based on Bonaparte et al. 2001, Soares et al. 2011).

**Character 21:** *Riograndia* changes from ? to 1 (based on Bonaparte et al. 2001, Soares et al. 2011).

**Character 22:** *Riograndia* changes from ? to 0 (based on Bonaparte et al. 2001, Soares et al. 2011).

**Character 26:** *Riograndia* changes from ? to 0 (based on Soares et al. 2011).

**Character 27:** *Riograndia* changes from ? to 1 (based on Bonaparte et al. 2001).

**Character 30:** *Prozostrodon* changes from ? to 2.

**Character 35:** *Brasilodon* changes from 2 to 1 (based on Bonaparte et al. 2005).

**Character 37:** *Brasilodon* changes from ? to 1 (based on Bonaparte et al. 2005).

**Character 80:** *Riograndia* changes from ? to 0 (based on Bonaparte et al. 2001, Soares et al. 2011).

**Character 93:** *Prozostrodon* changes from 0 to 1 (based on UFRGS-PV-0248-T).

**Character 96:** *Exaeretodon* and *Scalenodon angustifrons* change from 1 to 0 (due to change in character definition).

**Character 98:** *Brasilodon* changes from ? to 0.

**Character 102:** *Probainognathus* changes from 1 to 0.

**Character 103:** *Riograndia* changes from 1+2 to 1 (based on Bonaparte et al. 2001, Soares et al. 2011). *Prozostrodon* changes from 0 to 1.

**Character 106:** *Adelobasileus* changes from 1 to ?.

**Character 112:** *Adelobasileus* changes from 0 to ?.

**Character 115:** *Probainognathus* changes from 0 to 1. *Adelobasileus* changes from 0 to ?.

**Character 119:** *Brasilodon* changes from 0 to 0+1.

**(3) Data Matrix**

TNT file of the data matrix corresponding to the data set analyzed in this paper. Inapplicable characters are marked with a dash.

xread

145 35

Procynosuchus

0000000100000000000000000000000000000000000000000000000000000001[01]00000000000000000000001000000000100010000000--00-0000000000000-00000000000000002

Galesaurus

0100000000000000001000001000011000000000000000000000000000000000000000000000010000100000000001100100000010000--00-1000000001100????00??0??0000000

Thrinaxodon

01[01]0000000000000000000000000001110000000000000000000000000000000000000001001010000100000110001100000010000000--00-1000000001100-00000000000?0000?

Platycraniellus

0110000000000000001100000000010110000000??00000000?0000000000000000000101??101000?1010?0??0001?0?100000000000--0?-0?000??????????????????????????

Cynognathus

00000000000000001021002110000011100001011100010100?0000000000000100000111001011011201011120001101000100010000--00-1001000000111000000110000000000

Diademodon

[01]0000000000000001022012111000111100000011100010100000000[01]000000000000000100101111120101112113110100012001000100100?000100001111000000110000000000

Trirachodon

11100100[01]00000001121011111010011101002011100010100000002100000000000110111020110111010111211311010001211100020110100101???01111000000????00000000

Sinognathus

1020?100100000??11010011110?1?1?1010?0011000010100???????00000?000?011011102011011101011??113120000002?0?00?201100?0201??????????????????????????

Langbergia

001000000000000001210111110100111010000110000101?0?000?21??0000000001?0?11????10111010111211311010001200100020110100101?????1????????????????????

Pascualgnathus

1020?100100000001122012111011?1110100001??000101?0???0?2?00000???0?0??????????[01]0?1201011??113210000002[12][12]000-110100-12010???1101??0000??1000000000

Luangwa

??00?1000?0000010121001111????1110?00001???00101000000?2??00000???0???????????1011201011??113110100012[12][12]000-202100-12010???110110000???1100000000

Massetognathus

0111110010000000110101111101121110200001000001010000001211000000000011011102011011[12]01011??11311021110211000-222100-120100000101101000??1100000000

Exaeretodon

00111110100000111121012111011211101[01]00010?00010100010002110000000000?101????011011211011??113210010102[12][12]000-120100-122100000001101001111100000000

Scalenodon

??10?1?0000000??1101012111?????1101?0??1??00010100?0000211000000?000??0?????0?[01]0?12??011??113110100012[12][12]000-202100-1201??????????????????????????

Mandagomphodon

???0010?????001?11?????111011211101?0?0?0??00????????????1?000?0?????1??1?0?0?[01]??1???011??113220001002[12][12]000-222100-1201??????????????????????????

Chiniquodon

11101010100000101011000001011[12]11112100011000010100000001000000000000?1?01???111011201011120001100000000010000--00-100000??000011010001110000?0000

Lumkuia

??1000101000000?00000000010?1201101001010100010010?0000001000000000000001000011011201000120001100000000010000--00-10000?????001????????????0?????

Ectenion

001??0021000002000000000000?1[01]11100002011100010100?1000101000000000011001102111011001011??00011000001[01]0010000--00-?00000???1??110??0?????0???????

Probainognathus

0110100210000010010110000101121111100001110001000000001100000000000011001102111021001011120001100000000000000--00-10110000?000110???0??11000?0000

Bonacynodon

??1??0?21??000??01011000?10??211111000?????0?????0????????????0??000???????????0?0001?1112?001?0000?100000000--00-?0110??????????????????????????

Therioherpeton

?????0121?11122?2100??0????????1111???????????????????????????????????????????????????????000????????0?0?1100--00-?00000??1?00?????????1111110011

Protheriodon

??0??0??????????20001?0???????011110???????????????????????????????????????????0?1?0??11?????100001?00?000000--00-?010???????????????????????????

Riograndia

20131012111112212100100000111201112120110001020000?0000102000010001121102?13???000301111??0012110011001001100--00-10100??????????????????????????

Pachygenelus

20131012111112212100100000011201112120110001020000100001020000101??121102213132020301111120012210010001000010--00-002001???0001111101??1111111111

Prozostrodon

21?010?2????1221?1?????????112?1111?1????????????????????????????????????????????0301111??0010000000111001100--00-001000???000?????0???1111110000

Botucaraitherium

?????0???????????????????????????????????????????????????????????????????????????0???11????01??????001?001110--01-00??0??????????????????????????

Brasilodon

[01]000?0121121122120001000?00?1201111112110001021001?0?112220?00111011211022131320?0301111??0011100001011001110--01-001[01][01]??????0111??11????????????

Brasilitherium

0000?0121121122120001000000?1201111102110001021011?01112220???11?01121102213132010301111??0010100001011001110--01-001[01]1????????????11????????1111

Tritylodon

102-111111011221110200111101121110212211000011011011010212111101101031102203120200311111??1132210-22-222-32-2-1100-03221??????210??????1?????1111

Oligokyphus

[12]??-1111?10112???102010110?1?2?1??21????????110?10110102?2111101100031002203120200311111??1132110-22-222-32-2-1100-0322111100?2101111??1121111111

Bienotherium

102-11111101122111?201?111011211102122110000110110110?02?01111?110?031??22131?0200311111??1132110-22-222-32-2-1100-03221??????210??11??????111111

Kayentatherium

102-11111?0112211102011111111201102122110000110110110102121111?110?031102213120200311111121132110-22-222-32-2-1100-0322?11100021011?1111121??1111

Adelobasileus

???????01121?2????????????????0??????2110001021011?21112210000101110?????????????????????????????????????????????????????????????????????????????

Sinoconodon

0002?0101121122120001000?01?1211112102110011031011?21112221011101010????????0?3020301112??2001000001001002210--00-10101??????????????????????????

Morganucodon

0?02?0101121122120002?000011111111210211001103201112111222011211111121102213243020301112122221000001011002210--01-0010111111001111111??1121111111

;

ccode [-.;

proc/;

**(4) Synapomorphies**

List of synapomorphies in the probainognathian clade for the strict consensus tree (Figure 18). Unambiguous character-states have an asterisk (*).

Probainognathia: 56(1)*, 69(1), 70(1), 74(1), 76(2), 77(1)*

*Chiniquodon* + Probainognathidae + Prozostrodontia: 30(2), 34(1)*, 124(0)

Probainognathidae + Prozostrodontia: 21(1)*, 48(0), 81(2)*, 105(0), 117(1), 137(1)

Probainognathidae: 118(1)

Prozostrodontia: 17(2)*, 31(0)

*Prozostrodon* + more inclusive clade: 82(0), 106(1)*, 107(1)*

*Therioherpeton* + more inclusive clade: 144(1)*, 145(1)*

Tritheledontidae + more inclusive clade: 35(2), 120(1)*, 142(1)*, 143(1)*

Tritheledontidae: 4(3)*, 68(1), 99(1)

Tritylodontidae + *Botucaraitherium* + more inclusive clade: 38(2), 49(1), 54(1)*, 56(2), 132(1)*, 138(2)*

Tritylodontidae: 6(1), 8(1), 20(2), 21(0), 22(1), 24(1), 25(1), 45(1)*, 52(1), 59(1), 60(1), 61(1), 62(1), 69(3)*,78(2)*, 79(0), 80(2)*, 81(0), 84(1), 91(1), 92(1), 93(3), 99(2)*, 100(2)*, 103(2)*, 104(2)*, 106(3)*, 107(2), 109(2), 112(1), 117(3)*, 118(2), 119(2)*, 127(2)*

*Kayentatherium* + more inclusive clade: 23(1), 26(1)

*Tritylodon* + *Bienotherium*: 31(1)

*Botucaraitherium* + more inclusive clade: 113(1)*

*Brasilodon* + *Brasilitherium* + Mammaliaformes: 100(1), 119(1)

*Brasilodon* + *Brasilitherium*: 35(1), 66(1)

Mammaliaformes: 8(0), 52(2)*

*Sinoconodon* + *Morganucodon*: 31(1), 43(1)*, 46(3)*, 61(1)

**References**

Bonaparte J F, Barberena MC. On two advanced carnivorous cynodonts from the Late Triassic of Southern Brazil. Bull Mus Comp Zool. 2001;156:59–80.

Bonaparte JF, Ferigolo J, Ribeiro AM. A primitive Late Triassic “ictidosaur” from Rio Grande do Sul, Brazil. Palaeontology. 2001;44:623–635.

Hopson JA, Kitching JW. A probainognathian cynodont from South Africa and the phylogeny of nonmammalian cynodonts. Bull Mus Comp Zool. 2001;156:5–35.

Liu J, Olsen PE. The phylogenetic relationships of Eucynodontia (Amniota, Synapsida). J Mammal Evol. 2010;17:151–176.

Martinelli AG, Bonaparte JF, Schultz CL, Rubert R. A new tritheledontid (Therapsida, Eucynodontia) from the Late Triassic of Rio Grande do Sul (Brazil) and its phylogenetic relationships among carnivorous non-mammalian eucynodonts. Ameghiniana. 2005;42:191–208.

Martinelli AG, Rougier GW. On *Chaliminia musteloides* Bonaparte (Cynodontia, Tritheledontidae) and the phylogeny of the Ictidosauria. J Vertebr Paleont. 2007;27:442–460.

Soares MB, Schultz CL, Horn BL. New information on *Riograndia guaibensis* Bonaparte, Ferigolo & Ribeiro, 2001 (Eucynodontia, Tritheledontidae) from the Late Triassic of southern Brazil: anatomical and biostratigraphic implications. An Acad Bras Ciênc. 2011;83:329–354.

Soares MB, Martinelli AG, Oliveira TV. A new prozostrodontian cynodont (Therapsida) from the Late Triassic *Riograndia* Assemblage Zone (Santa Maria Supersequence) of Southern Brazil. An Acad Bras Ciênc. 2014;86(4):1673–1691.
